# Does facility birth reduce maternal and perinatal mortality in Brong Ahafo, Ghana? A secondary analysis using data on 119 244 pregnancies from two cluster-randomised controlled trials

**DOI:** 10.1016/S2214-109X(19)30165-2

**Published:** 2019-07-11

**Authors:** Sabine Gabrysch, Robin C Nesbitt, Anja Schoeps, Lisa Hurt, Seyi Soremekun, Karen Edmond, Alexander Manu, Terhi J Lohela, Samuel Danso, Keith Tomlin, Betty Kirkwood, Oona M R Campbell

**Affiliations:** a Heidelberg Institute of Global Health, Heidelberg University, Heidelberg, Germany; b Faculty of Epidemiology and Population Health, London School of Hygiene and Tropical Medicine, London, UK; c Research Department 2, Potsdam Institute for Climate Impact Research, Potsdam, Germany; d Institute of Public Health, Charité – Universitätsmedizin Berlin, Berlin, Germany; e Observational and Pragmatic Research Institute, Singapore; f Division of Population Medicine, Cardiff University School of Medicine, Cardiff, UK; g Faculty of Life Sciences and Medicine, King's College London, London, UK; h Kintampo Health Research Centre, Kintampo, Ghana; i Liverpool School of Tropical Medicine, Liverpool, UK; j Department of Public Health, University of Helsinki, Helsinki, Finland; k University of Edinburgh Medical School, Edinburgh, UK

## Abstract

**Background:**

Maternal and perinatal mortality are still unacceptably high in many countries despite steep increases in facility birth. The evidence that childbirth in facilities reduces mortality is weak, mainly because of the scarcity of robust study designs and data. We aimed to assess this link by quantifying the influence of major determinants of facility birth (cluster-level facility birth, wealth, education, and distance to childbirth care) on several mortality outcomes, while also considering quality of care.

**Methods:**

Our study is a secondary analysis of surveillance data on 119 244 pregnancies from two large population-based cluster-randomised controlled trials in Brong Ahafo, Ghana. In addition, we specifically collected data to assess quality of care at all 64 childbirth facilities in the study area. Outcomes were direct maternal mortality, perinatal mortality, first-day and early neonatal mortality, and antepartum and intrapartum stillbirth. We calculated cluster-level facility birth as the percentage of facility births in a woman's village over the preceding 2 years, and we computed distances from women's regular residence to health facilities in a geospatial database. Associations between determinants of facility birth and mortality outcomes were assessed in crude and multivariable multilevel logistic regression models. We stratified perinatal mortality effects by three policy periods, using April 1, 2005, and July 1, 2008, as cutoff points, when delivery-fee exemption and free health insurance were introduced in Ghana. These policies increased facility birth and potentially reduced quality of care.

**Findings:**

Higher proportions of facility births in a cluster were not linked to reductions in any of the mortality outcomes. In women who were wealthier, facility births were much more common than in those who were poorer, but mortality was not lower among them or their babies. Women with higher education had lower mortality risks than less-educated women, except first-day and early neonatal mortality. A substantially higher proportion of women living in areas closer to childbirth facilities had facility births and caesarean sections than women living further from childbirth facilities, but mortality risks were not lower despite this increased service use. Among women who lived in areas closer to facilities offering comprehensive emergency obstetric care (CEmOC), emergency newborn care, or high-quality routine care, or to facilities that had providers with satisfactory competence, we found a lower risk of intrapartum stillbirth (14·2 per 1000 deliveries at >20 km from a CEmOC facility vs 10·4 per 1000 deliveries at ≤1 km; odds ratio [OR] 1·13, 95% CI 1·06–1·21) and of composite mortality outcomes than among women living in areas where these services were further away. Protective effects of facility birth were restricted to the two earlier policy periods (from June 1, 2003, to June 30, 2008), whereas there was evidence for higher perinatal mortality with increasing wealth (OR 1·09, 1·03–1·14) and lower perinatal mortality with increasing distance from childbirth facilities (OR 0·93, 0·89–0·98) after free health insurance was introduced in July 1, 2008.

**Interpretation:**

Facility birth does not necessarily convey a survival benefit for women or babies and should only be recommended in facilities capable of providing emergency obstetric and newborn care and capable of safe-guarding uncomplicated births.

**Funding:**

The Baden-Württemberg Foundation, the Daimler and Benz Foundation, the European Social Fund and Ministry of Science, Research, and the Arts Baden-Württemberg, WHO, US Agency for International Development, Save the Children, the Bill & Melinda Gates Foundation, and the UK Department for International Development.

## Introduction

Annually, more than 1 million newborn babies die on the day they are born^1^, ^2^ and 1·3 million stillbirths occur during labour and birth,^3^ which is also when 46% of maternal deaths occur.^1^ Acknowledging these epidemiological facts has led to the prioritisation of intrapartum care,^4^ namely birth with a skilled attendant and in a health facility.^5^ However, empirical evidence for the benefits of facility birth is scant,^6^ and has only started to emerge, with ambiguous findings. Effect estimates have been largely based on a single before–after study from Bangladesh^7^ and on expert opinion.^8^, ^9^, ^10^, ^11^ Moreover, the extent to which facility birth can translate into mortality decline crucially depends on the quality of care provided. A substantial body of evidence is emerging that documents low provider skills and limited facility capability to provide good-quality routine and emergency care at birth.^12^ This evidence might explain the mismatch between high coverage of facility birth and persistently high mortality burdens in many settings.^13^

**Research in context**

**Evidence before this study**

We searched PubMed on Aug 28, 2018, without any language restrictions for all articles in which the title or abstract contained the search terms “pregnancy-related death”, “pregnancy-related mortality”, “maternal mortality”, “maternal death”, “neonatal mortality”, “neonatal death”, “stillbirth”, “perinatal death”, or “perinatal mortality”, and “skilled birth attendant”, “skilled birth attendance”, “health professionals”, “institutional delivery”, “institutional deliveries”, “facility-based delivery”, “facility-based deliveries”, “obstetric care”, “distance”, or “facility delivery”. Reference lists of the included studies were searched to identify other relevant studies. Most studies on the effect of facility birth on mortality focused on one mortality outcome (maternal, neonatal, or stillbirth) and used one of three approaches: individual women's place of delivery or type of attendant at birth; aggregated measures of facility birth at the country, district, or village level; and distance as a measure of access to health care.

The first approach was used frequently although it is highly problematic because facilities attract women with complications, and these women and their babies are more likely to die, leading to confounding by case mix. Studies using aggregate measures, mostly ecological studies, show that greater use of facility birth at country level is linked to lower mortality; however, health systems and income levels and other determinants linked to mortality outcomes also differ between countries, and might confound the association. There are few studies using aggregate measures at subnational level, and these studies have mixed findings. Evidence that shorter distance from a childbirth facility is linked to lower mortality is sparse, with widely differing results between studies and settings. Furthermore, under-reporting and misclassification of deaths in cross-sectional surveys is a concern, and most studies on the topic did not have sufficient information on the quality of care provided in facilities. Several reviews and meta-analyses have been done, but with contradictory and inconclusive results, no doubt in part because they included studies with inadequate methods.

**Added value of this study**

To the best of our knowledge, this study is the first to examine the effect of facility birth on birth-related mortality comprehensively, using high-quality prospectively collected data from a large population-based cohort. We studied the effects of cluster-level facility birth (percentage of facility births in a woman's village over the 2 calendar years preceding the index birth), household wealth, education, and distance to care on a comprehensive set of mortality outcomes, comprising direct maternal mortality, antepartum and intrapartum stillbirth, overall stillbirth, first-day and early neonatal mortality, and perinatal mortality, as well as on facility birth and caesarean section. We also studied the effect of distance to high-quality facilities on mortality, considering several quality dimensions. Furthermore, we assessed the effect of policy changes towards free childbirth care that increased facility birth and potentially led to overcrowding and a deterioration in quality of care.

We found that proximity to the closest facility offering childbirth care (of any quality) and household wealth substantially increased facility birth, but did not decrease mortality of women or babies. Living in a village where facility birth was more common was also not linked to lower mortality. Surprisingly, closer distance to a facility offering high-quality care at birth did not reduce neonatal or maternal mortality, but did reduce the risk of intrapartum stillbirth. We found that facility birth was associated with higher mortality in the most recent time period, suggesting that the policy shift might have compromised quality of care. We thus provide crucial evidence on the importance of quality of care at birth to achieve reductions in mortality.

**Implications of all the available evidence**

In settings with low facility capability, giving birth in a facility does not confer any survival benefit for women or babies. This does not mean we should stop recommending birth with a skilled attendant, including in facilities. Rather, we should ensure that all health facilities fulfil their requirements and are actually capable of providing life-saving emergency obstetric and newborn care, and providing good care for uncomplicated, physiological births. Birth attendants also need competency-based training to ensure they are actually skilled. Policies to increase care-seeking should be accompanied by proper planning and financing to ensure that quality can be maintained or enhanced. The focus should shift from just increasing coverage of facility birth to improving quality of care and to developing appropriate metrics to track this progress.

The important question on the extent to which facility birth decreases mortality in different contexts is methodologically challenging to answer. Individual-level studies on the link between facility birth and mortality are rarely interpretable because adverse selection leads to confounding by case mix. Women who have complications in pregnancy or during childbirth are more likely to seek care at health facilities, and they and their babies are also more likely to die.^6^ In addition, it is difficult to measure and adjust for complications and their severity well enough and in the same way for home and facility births. Evidence of substantial declines in health facility mortality, used historically to argue for facility birth in high-income countries, is legitimately contested, because increases in institutional births brought more low-risk deliveries into facilities (ie, changed the case mix).^14^

Reviews of individual-level studies have led to inconclusive results.^6^, ^8^, ^15^, ^16^ An analysis of place of birth and neonatal mortality in 192 Demographic and Health Surveys (DHS) from 67 low-income and middle-income countries found significantly lower mortality among facility births than home births in 16 countries, significantly higher mortality in ten countries, and an overall null effect (adjusted odds ratio [OR] 1·00, 95% CI 0·97–1·03).^17^

In principle, the problem of individual-level studies can be avoided by: studying facility birth at an aggregate level; studying the mortality effects of a major determinant of facility birth, such as wealth, education, or distance; or studying policy changes that affect access to facility birth and potentially also quality of care. We can also study quality of care more explicitly, by refining analyses of the effect of distance on mortality by using distance to facilities that provide certain standards of obstetric or neonatal care.

Applying the first (aggregate-level) approach via ecological studies of countries usually shows that countries with higher percentages of health facility births have lower maternal and perinatal mortality than countries with lower percentages of facility births.^6^, ^18^, ^19^ However, these results are difficult to accept confidently, given that countries differ in their health systems and income levels.^6^ A better approach is to examine the association using subnational units, such as districts or settlements,^6^ which has been done in a few studies.^20^, ^21^, ^22^, ^23^ An ideal aggregate approach would use facility birth in previous years on the aggregate level as a predictor for individual-level mortality outcomes, thus allowing adjustment for individual-level confounders while avoiding confounding by case mix.

The second approach is to study the association between a determinant known to increase use of facility birth, such as wealth, education, or distance to childbirth care, and mortality (at the population level, not among users of health facilities). There is strong and abundant evidence that higher household wealth and maternal education increase facility birth,^24^, ^25^ whereas the evidence that they reduce early neonatal mortality or stillbirth is inconsistent.^26^, ^27^, ^28^, ^29^ An analysis of distance to services in 29 DHS datasets showed a significant increase in neonatal mortality with increased distance in the pooled sample, but suggested that neonatal mortality was lower at increased distance in nine countries, significantly so for Nigeria.^30^ These inconsistent results are exacerbated by concerns about differential under-reporting of deaths or misreporting of early neonatal deaths as stillbirths in some DHS.^31^

To date, few studies on facility birth and mortality have assessed the capability of childbirth facilities to provide good-quality obstetric and neonatal care,^27^, ^32^ either directly or indirectly by studying the effect of policy changes that affect quality of care. In Ghana, free childbirth care was implemented in 2005, followed by free health insurance for pregnant women in 2008.^33^ These policy shifts rapidly increased facility birth, and reduced socioeconomic inequalities in facility use.^33^ Facility resources were not increased concomitantly, which overstretched health workers, and might well have compromised quality of care.^34^

This Article assesses the effect of facility birth on maternal and perinatal mortality in Ghana with prospectively collected population-level data from two large-scale trials and a detailed Health Facility Assessment of quality of care in seven districts, using a number of valid methodological approaches. Our specific objectives are to quantify the associations of cluster-level facility birth, household wealth, mother's education, distance to any childbirth care, and distance to high-quality childbirth care on maternal and perinatal mortality, and to study how these associations vary over time periods reflecting policy change in Ghana. If facility care at birth is effective (of good quality), then mortality should be lower among population subgroups that are more likely to use services than among those that are less likely to use services, except possibly for antepartum stillbirth, which is less affected by care at birth.

## Methods

### Setting and outcome variables

Our study is a secondary analysis of data from two cluster-randomised controlled trials, ObaapaVitA^35^ and Newhints,^36^ for which data were continuously collected between 2000 and 2009 in seven contiguous districts of the Brong Ahafo region in Ghana. ObaapaVitA^35^ tested the effect of low-dose vitamin A supplementation on mortality of women of reproductive age (enrolled at age 15–45 years) and of their babies, and collected data from Dec 11, 2000, enrolling women in a staggered way across districts, until Oct 31, 2008. Newhints^36^ tested the effect of home visits by community-based surveillance volunteers on neonatal mortality, and collected data from Nov 1, 2008, to Dec 31, 2009. Neither study showed a significant effect on mortality.^35^, ^36^ The surveillance system established for the trials included home visits every 4 weeks to women of reproductive age to identify and register pregnancies, births, and deaths. Data were collected on place of delivery, caesarean section, pregnancy-related mortality, stillbirth, and neonatal mortality, as well as sociodemographic characteristics. Data collection is described in the key trial publications.^35^, ^36^

We harmonised and jointly analysed data from the ObaapaVitA^35^ and Newhints^36^ trials. The unit of all analyses was the delivery episode (including deaths in women who had not delivered), which meant a woman could contribute several delivery episodes over time and that twin or triplet births were considered as one episode. A delivery episode was considered to result in stillbirth or early neonatal death if at least one baby fulfilled the criteria for this outcome, so in a few cases, a delivery episode was counted as having resulted in two different outcomes (eg, if twins died at different timepoints). Births in hospitals, health centres, clinics, or maternity homes were considered to be facility births.

The mortality outcomes we considered were: stillbirth (born dead after at least 6 months of gestation), separated into antepartum and intrapartum stillbirth (further details available in the study by Ha and colleagues);^37^ early neonatal death (death of a liveborn infant within the first 7 days of delivery), with the subgroup first-day neonatal death (death of a liveborn infant within 24 h of delivery); perinatal death (stillbirth or early neonatal death); and direct maternal death (death from obstetric complications or interventions during pregnancy or within 42 days thereof). Livebirths with incomplete follow-up for the first 7 days were excluded from the analyses of early neonatal and perinatal mortality.

### Determinants of facility birth

Cluster-level facility birth was calculated as the percentage of facility births in a village or suburb. We used cluster-level facility birth in the preceding 2 calendar years as a predictor for the index birth. Unlike using births in the same year, this strategy avoids confounding by complications at the cluster level. Some delivery episodes from a few very small villages were excluded from this analysis because they had no births recorded in the preceding 2 years, leading to missing values in cluster-level facility birth. The same is true for births before 2003, when no childbirth records of the previous 2 calendar years were available.

To measure wealth, we calculated household asset quintiles using principal component analysis of household assets according to DHS methodology.^38^ Mother's education was coded in four levels: none; primary school; middle school or junior secondary school; and technical, commercial, or senior secondary school, or post-middle college, or post-secondary or higher education.

We used global positioning system coordinates of health facilities and village centroids to calculate distances from the woman's regular place of residence to the closest health facility and to the closest high-quality health facility, considering several quality dimensions. Straight-line distances to a comprehensive emergency obstetric care (CEmOC) facility ranged from less than 1 km to 84 km.^39^ Women in three of the larger towns (Nkoranza, Techiman, and Kintampo) were assigned the centroid of the respective suburbs as their place of residence. Road network data were used to calculate road distance and travel-time measures for sensitivity analyses.^39^

### Quality of care at health facilities

For the purpose of this analysis, we visited all 86 health facilities in the study area in 2010 to assess quality of obstetric and newborn care. Of the 64 facilities offering childbirth care, 24 were classified as capable of providing high-quality routine care, 12 as capable of providing emergency obstetric care (EmOC), of which eight were capable of providing CEmOC, and five were capable of providing emergency newborn care (EmNC).^40^ Detailed information on methods and findings of this comprehensive health facility assessment have been published elsewhere.^40^, ^41^ Briefly, we used information on key signal functions, availability of drugs, equipment, and trained health professionals to create quality scores of different dimensions of care, including routine childbirth care, CEmOC, and EmNC.

Furthermore, we used clinical vignettes to assess health professional competence, interviewing the most experienced provider, present at the day of visit, who manages childbirth and newborn infants at the facility. Two vignette cases tested ability to diagnose and manage conditions that threatened the lives of both mother and baby—pre-eclampsia and severe antepartum haemorrhage. On average, providers mentioned 11 of 20 necessary actions correctly, with the number of correct answers ranging from one to 15.^41^

The four quality-of-care variables used in this analysis were distance to the closest health facility offering CEmOC, distance to the closest facility offering EmNC, distance to the closest facility offering high-quality routine childbirth care, and distance to the closest facility with staff who achieved a vignette score of at least 12 of 20.

### Policy change

To assess the effect of Ghana's 2005 policy on free childbirth care and its 2008 policy on free national health insurance for pregnant women, we studied the association between facility birth and mortality during three time periods, defined in previous analyses.^33^ The first period reflected the time before the policy change, starting June 1, 2003 (because variables that were needed to adjust for confounding were consistently collected from this date) and finishing March 31, 2005. The second period started on April 1, 2005, when the nationwide delivery fee exemption policy was introduced, and finished June 30, 2008. The third period started on July 1, 2008, when free national health insurance was introduced for pregnant women; this period ended with the end of Newhints surveillance on Dec 31, 2009.^36^

### Statistical analysis

Although data on stillbirth, early neonatal mortality, first-day neonatal mortality, and perinatal mortality were available for the full sample (2000–09), data on antepartum stillbirth and intrapartum stillbirth were available only from June, 2003, to October, 2008, and data on direct maternal mortality only until the end of the ObaapaVitA trial^35^ in October, 2008. The total numbers of pregnancies, deliveries, and deaths in adjusted and unadjusted analyses are shown in a flowchart (appendix, p 2).

**Figure 1:**
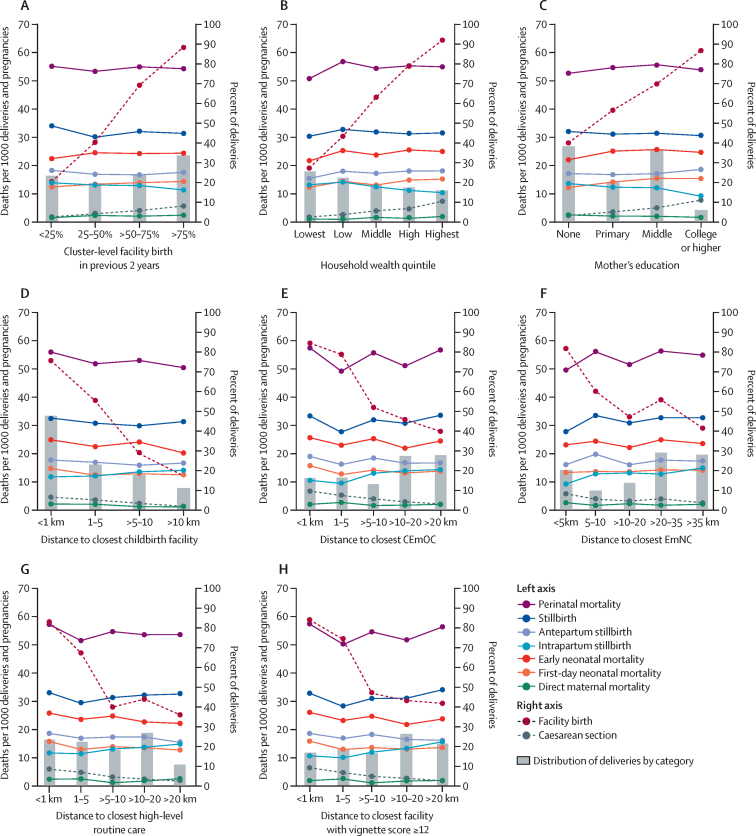
Health service use and mortality outcomes by cluster-level facility birth, wealth, education, and distance to facilities offering various levels of care Facility birth and caesarean section (right axis), and mortality (left axis) by cluster-level facility birth (A), household wealth (B), mother's education (C), distance to closest childbirth facility of any level (D), distance to closest facility providing CEmOC (E), distance to closest facility providing EmNC (F), distance to closest facility offering high-level routine care (G), and distance to closest facility with satisfactory provider competence (H). CEmOC=comprehensive emergency obstetric care. EmNC=emergency newborn care.

For presentation in figure 1, we categorised continuous exposure variables into a small number of groups, so that the proportion of facility births, caesarean sections, and all types of mortality risks could be plotted by category. We then assessed associations in crude and multivariable two-level logistic regression models, with village of residence at level two, thus taking the similarities of births from the same village into account.

We analysed the effect of the proportion of cluster-level facility birth in the preceding 2 years as a continuous variable. The effects of household wealth were estimated per wealth quintile and those of mother's education were estimated per highest education level reached. To establish the functional shape of the association between distance and outcomes, we used fractional polynomials of first degree, assuming a monotone dose-response relationship.^42^ Across associations we found that transformations with slopes that flatten for larger distances, such as the logarithm or the square root of distance, were better than linear or quadratic slopes. Thus, all distance variables were log-transformed for the analyses.

Multivariable analyses were adjusted for year of birth, multiple birth, mother's age, parity, religion, ethnicity, occupation, education, wealth, and distance to closest CEmOC (in the models with wealth and education as main exposures) and restricted to births after June 1, 2003, because different data collection procedures before that date led to more missing values for adjustment variables. We then dropped observations with missing values in any of the adjustment variables, which amounted to about 1% of the sample after June 1, 2003.

The direct maternal mortality outcome was rare, with only 200 deaths during the entire observation period, and we wished to use all pregnancies from the year 2000 onwards, despite missing data on household wealth, education, occupation, and multiple birth for many women who died before 2003. We used multiple imputation (mi command in Stata) with 20 imputations for these four variables in an imputation model that included year of birth, mother's age, parity, religion, ethnicity, and the respective main exposure and the outcome variables. Thus, the regression models for direct maternal mortality were adjusted for year of birth, mother's age, parity, religion, ethnicity, occupation (partly imputed), education (partly imputed), wealth (partly imputed), multiple birth (partly imputed), and distance to the closest CEmOC (in the models with wealth and education as main exposures). This and all other analyses were done with Stata IC 14 software.^43^

For completeness and comparability to other studies, we also examined the association between individual-level facility birth and mortality outcomes in adjusted analyses (appendix, p 6). We also did four sensitivity analyses that are described and summarised in the appendix: crude analyses in the restricted sample from June, 2003 (appendix, p 14), using road distance and travel time (appendix, p 18), restricting the sample to women with good pregnancy surveillance (appendix, p 20) and using a three-level random-effects model (appendix, p 23). Results were very similar to the main results presented herein.

We obtained ethical approval from the London School of Hygiene and Tropical Medicine in London, UK, and from the Kintampo Health Research Centre in Kintampo, Ghana. All participants of the ObaapaVitA^35^ and Newhints^36^ trials provided written informed consent on recruitment. Health workers provided written informed consent for the health facility assessment before the start of data collection.

### Role of the funding source

The funders of the study had no role in study design, data collection, data analysis, data interpretation, or writing of this report. The corresponding author had full access to all the data in the study and had final responsibility for the decision to submit for publication.

## Results

Over 9 years,^35^, ^36^ 119 244 pregnancies were recorded among 85 478 women. Information on direct maternal mortality was available for 102 853 pregnancies, of which 200 resulted in a direct maternal death (mortality risk 194 per 100 000 pregnancies). 113 547 deliveries were recorded, of which 3577 resulted in a stillbirth (mortality risk 31·5 per 1000 deliveries). Follow-up for at least 7 days was completed for 110 161 livebirths, in which 2614 early neonatal deaths occurred (mortality risk 23·7 per 1000 livebirths). Perinatal mortality risk for 113 452 deliveries with complete follow-up was 54·0 per 1000 deliveries. Of the 2355 deliveries that resulted in at least one stillbirth between June 1, 2003, and Oct 31, 2008, when timing of stillbirth was coded, 993 were intrapartum (mortality risk 12·5 per 1000 deliveries) and the remaining were antepartum. None of the mortality risks showed trends over time (appendix, p 5).

Facility birth increased from 1585 (36%) of 4402 births in 2001 to 9426 (69%) of 13 692 births in 2009, and hospital birth rose from 799 (18%) of 4402 births to 5843 (43%) of 13 692 births during the same period. Caesarean sections rose from 338 (4%) of 7719 births in 2003 (when mode of delivery was consistently collected) to 979 (7%) of 13 682 births in 2009. Cluster-level facility birth, household wealth, mother's education, and distance to closest childbirth facility were all strong determinants of facility birth and of caesarean section (figure 1). For instance, 15 005 (92%) of 16 337 births in the richest wealth quintile were in a facility, compared with only 7452 (27%) of 27 139 births in the poorest quintile (figure 1).

As anticipated, the problematic analysis of individual-level facility birth showed a strong (but biased) association with higher mortality for all studied outcomes, except maternal mortality, for which it did not differ (appendix, p 6).

**Figure 2:**
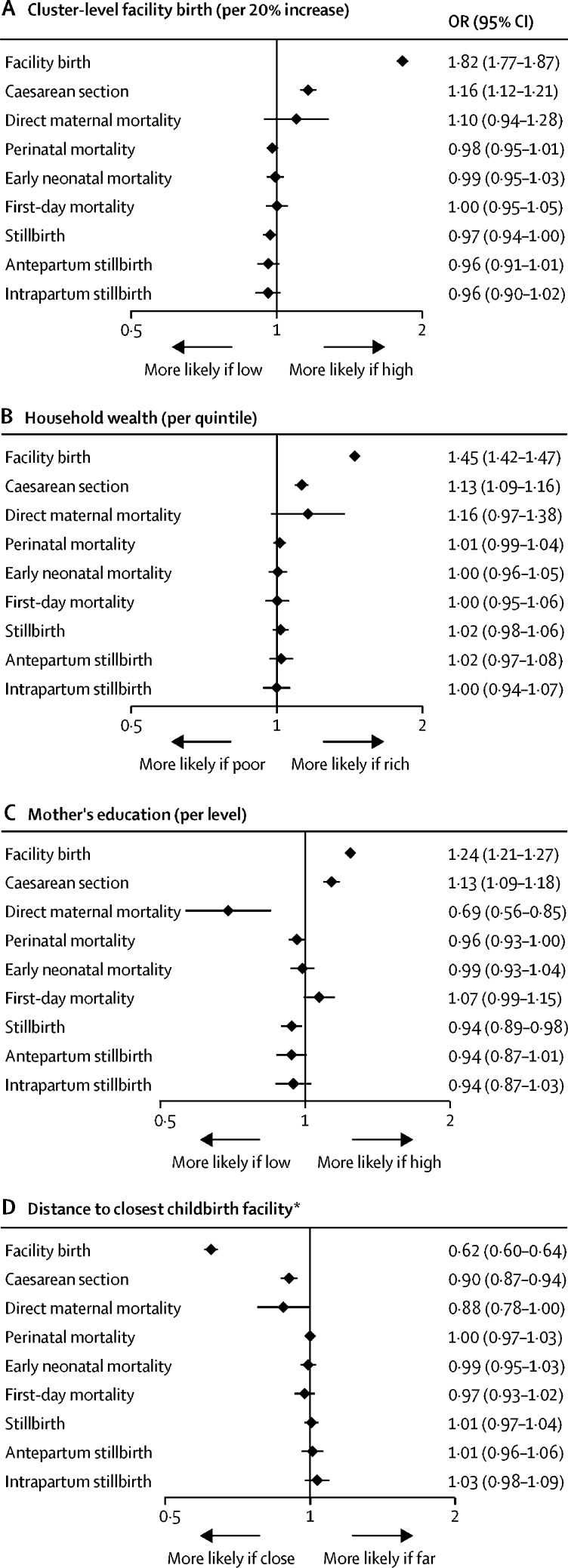
Adjusted effects of cluster-level facility birth (A), household wealth (B), mother's education (C), and distance to closest childbirth facility (D) on health service use and mortality We present ORs and 95% CIs from multilevel multivariable regression models adjusted for age, parity, religion, ethnicity, wealth, education, occupation, multiple birth, birth year, and (in the models with wealth and education as main exposures) distance to the closest comprehensive emergency obstetric care facility, using surveillance data from 2003 (from 2000 for maternal mortality) to 2009 (to 2008 for maternal mortality, antepartum stillbirth, and intrapartum stillbirth). Each panel shows the effects of an exposure on two health service use and seven mortality outcomes. OR=odds ratio. *ORs are per one unit increase in log distance (in km).

Cluster-level facility birth was not significantly associated with any of the mortality outcomes (figure 1, 2). Neither was household wealth associated with any of the mortality outcomes, with effect estimates close to the null value or higher (figure 1, 2). Women with higher education, however, had fewer maternal deaths and fewer stillbirths than women with lower education. This result was only clearly visible in adjusted analyses (figure 2) because of negative confounding by ethnicity and parity. Education level did not influence early neonatal mortality (figure 2). By contrast to the clear decline of facility birth and caesarean section with distance to the closest childbirth facility, longer distance to the closest facility (of any quality) was not associated with increased mortality of women or babies, either crudely (figure 1; appendix, p 7) or when adjusted for confounders (figure 2; appendix, p 10). Rather, the results suggested maternal mortality decreased as distance from the closest childbirth facility increased.

**Figure 3:**
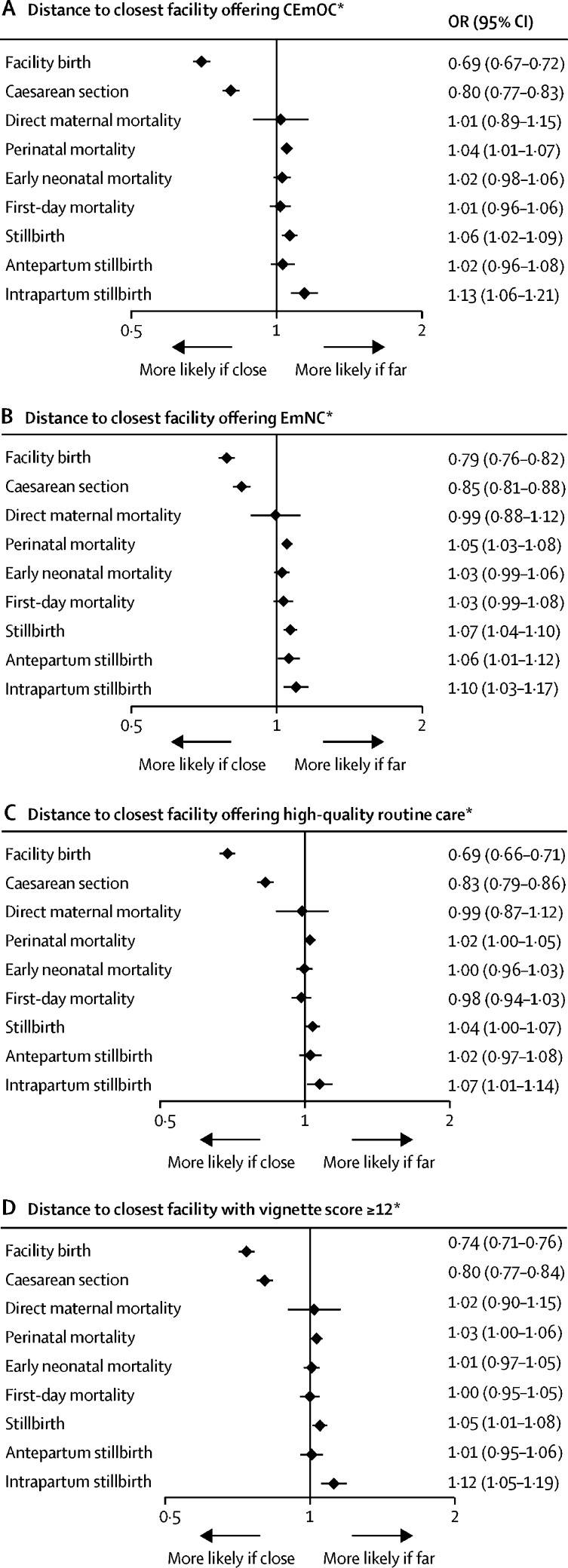
Adjusted effects of distance to the closest facility offering CEmOC (A), EmNC (B), high-quality routine care (C), and satisfactory provider competence (D) on health service use and mortality We present ORs and 95% CIs from multilevel multivariable regression models adjusted for age, parity, religion, ethnicity, wealth, education, occupation, multiple birth, and birth year, using surveillance data from 2003 (from 2000 for maternal mortality) to 2009 (to 2008 for maternal mortality, antepartum stillbirth, and intrapartum stillbirth). Each panel shows the effects of distance to a facility offering high-quality care in a certain dimension on two health service use and seven mortality outcomes. CEmOC=comprehensive emergency obstetric care. EmNC=emergency newborn care. OR=odds ratio. *ORs are per one unit increase in log distance (in km).

Longer distance to a facility offering high-quality care—namely CEmOC or EmNC, or a facility with satisfactory provider competence (vignette score ≥12 of 20)—was strongly associated with higher intrapartum stillbirth, which also led to significant associations for the composite outcomes of overall stillbirth and perinatal mortality in the adjusted analyses (figure 3; appendix, p 10). At more than 20 km from a CEmOC facility, where 40% of births were in a facility (and 10% in a CEmOC facility; data not shown), 14·2 intrapartum stillbirths occurred per 1000 deliveries, whereas within 1 km of a CEmOC facility, where 84% of births were in a facility (and 60% in a CEmOC facility; data not shown), only 10·4 intrapartum stillbirths occurred per 1000 deliveries (figure 1). In the adjusted model with log-transformed distance to CEmOC as a continuous exposure, the OR for intrapartum stillbirth was 1·13 (95% CI 1·06–1·21; figure 3) per one unit increase in log-transformed distance. The shape of the associations of service use and mortality outcomes with distance to CEmOC from the adjusted models is shown in the appendix (p 3). The results for distance to high-quality routine childbirth care were similar but slightly weaker than for the quality dimensions related to emergency care (figure 3). By contrast to intrapartum stillbirth, the outcomes maternal mortality and first-day or early neonatal mortality were not associated with distance to a facility offering high-quality care at birth in any of the dimensions (figure 1, 3).

**Figure 4:**
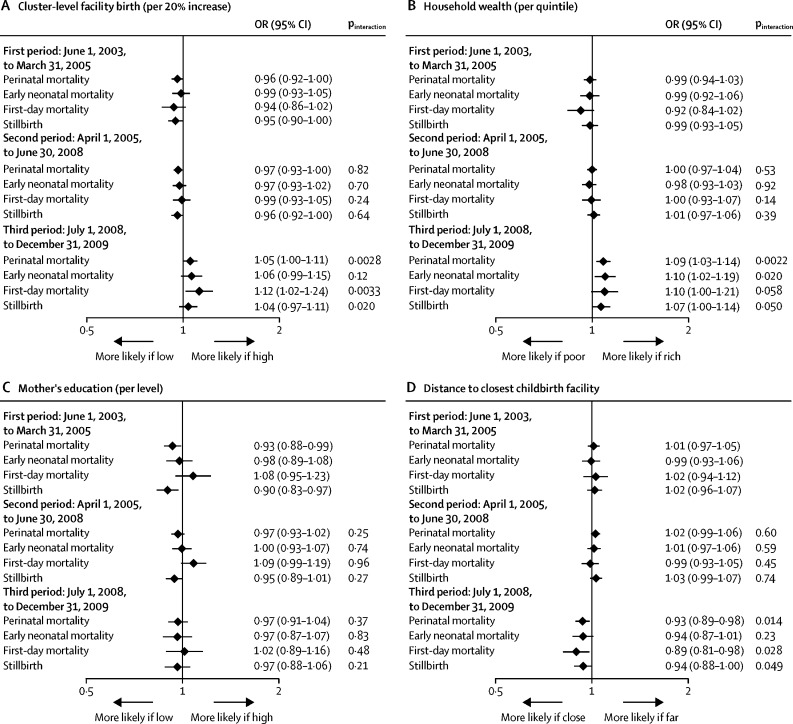

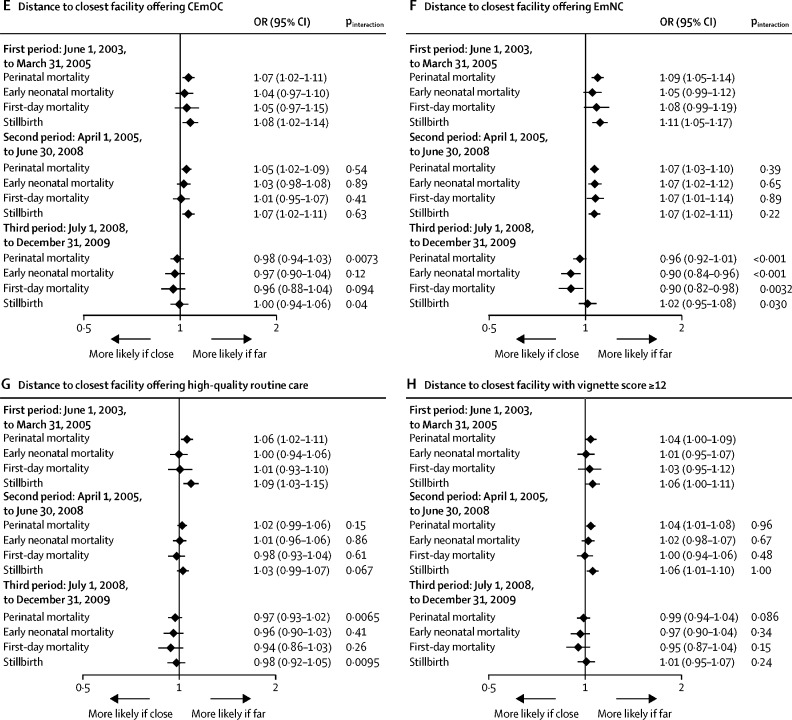
Effect modification of the association between facility birth and perinatal mortality by time period with different policies We present ORs and 95% CIs from multilevel multivariable regression models adjusted for age, parity, religion, ethnicity, wealth, education, occupation, multiple birth, birth year, and (in the models with wealth and education as main exposures) distance to the closest comprehensive emergency obstetric care facility, using surveillance data from 2003 to 2009. Each panel shows the effects of an exposure (cluster-level facility birth [A], household wealth [B], mother's education [C], distance to closest childbirth facility [D], and distance to closest facility offering CEmOC [E], EmNC [F], high-quality routine care [G], or satisfactory provider competence [H]) on perinatal mortality and its components, stratified by three time periods during which different policies were implemented. Interaction p values are given for the null hypothesis, being no difference in the exposure effects on mortality by time period (comparing the second to the first, and the third to the first period). Exposures are treated as continuous variables, and are continuous over categories for wealth and education. Effects are presented as a change in the odds of death per 20% increase in cluster-level facility birth, per one quintile increase in wealth, per one level increase in education, and per one unit increase in log distance (in km). CEmOC=comprehensive emergency obstetric care. EmNC=emergency newborn care. OR=odds ratio.

Facility birth increased from 52% in the first period before the policy change (June 1, 2003, to March 31, 2005) to 58% in the second period with free childbirth care (April 1, 2005, to June 30, 2008) and to 68% in the third period with free national health insurance (July 1, 2008, to Dec 31, 2009). When stratifying the aforementioned associations by time period, we found evidence that various predictors of higher facility birth were associated with higher perinatal mortality in the third time period (figure 4; appendix, p 13). Although in the first two periods perinatal mortality was lower in clusters in which facility birth was more common compared with those in which facility birth was less common, in the third period higher cluster-level facility birth was associated with higher perinatal mortality (p_interaction_=0·0028). Wealth showed no association with perinatal mortality in the first or second period, whereas in the third period, there was evidence that perinatal mortality increased with wealth (OR 1·09, 1·03–1·14; p=0·0014, p_interaction_=0·0022, for the third *vs* the first period). For education, there was no evidence for interaction by time period (p=0·37). Distance to the closest childbirth facility was not associated with perinatal mortality in the first or second period, whereas in the third period, perinatal mortality decreased with increasing distance from childbirth facilities (OR 0·93, 0·89–0·98; p=0·0069, p_interaction_=0·014, for the third *vs* the first period). Shorter distance to a facility offering CEmOC, EmNC, or high-quality routine care protected against perinatal mortality in the first period, and for CEmOC and EmNC was also protective in the second period, whereas there was no evidence for an association with perinatal mortality in the third period (interaction for third *vs* first period: p=0·0073 for CEmOC; p<0·001 for EmNC; and p=0·0065 for routine care). Neonatal mortality even decreased with longer distance from an EmNC facility in the third period. For the association between distance to a facility with satisfactory provider competence (vignette score ≥12) and perinatal mortality, no significant interaction by time period was observed (p=0·086).

## Discussion

Using data on 119 244 pregnancies in rural Ghana from the ObaapaVita^35^ and Newhints^36^ trials, we did not find evidence that facility birth decreased maternal or perinatal mortality. To avoid confounding by case mix, due to the fact that facilities attract more complicated births with higher mortality risks, we studied this link in different ways. We investigated the effect of cluster-level facility birth in the preceding years and other determinants known to influence facility use (wealth, education, and distance) on mortality, and in a further step, we considered the quality dimension by studying the effect of distance to facilities of different quality and by assessing the effect of a policy change that increased access but probably decreased quality of care.

Villages with high proportions of facility births had mortality risks that were similar to villages in which home birth was common. Wealthier women, as compared to women who were poorer, and those living closer to childbirth facilities, as compared to those further away, were much more likely to give birth in a facility. Nevertheless, mortality among these women or their babies was not any lower than among women from poorer households and those living far from facilities. In other words, certain population groups had substantially more facility births, but did not see corresponding survival gains. This finding suggests that facility birth by itself is not saving lives. Closer distance to facilities offering high-quality care at birth, however, was associated with a lower risk of intrapartum stillbirth and composite outcomes, but not of maternal or early neonatal mortality. Furthermore, we found that protective effects were restricted to the first two policy periods from June 1, 2003, to June 30, 2008, and we found evidence for higher perinatal mortality among facility births after free health insurance was introduced.

These sobering findings, interpreted in one way, risk undermining global strategies that encourage facility birth on the understanding that it can benefit all women—those with complications, those developing complications, and those with uncomplicated deliveries. However, we would rather emphasise that increasing facility birth does not translate into less mortality unless quality of care is assured and the gap between contact and content^44^ is closed. As highlighted previously, to bring women into a building with a health worker labelled as being skilled is not enough, but rather women should give birth in a health facility with good care that can save lives and prevent ill health.^12^, ^13^, ^45^ The repeated calls for a stronger focus on quality of care are underpinned by the evidence provided in this study of a reduction in intrapartum stillbirth only for the most capable facilities.

That intrapartum stillbirth is the outcome most closely aligned with care at birth fits with expectations that better access to CEmOC might prevent some of these deaths. Caesarean section, in particular, can prevent intrapartum stillbirth and be life saving for mother and baby if accessed in time, but the number of caesarean sections remains low in most sub-Saharan African countries.^46^ In our study population, the proportion of caesarean sections is higher than the minimum 5% only for certain subgroups, such as women living very close to a facility, or those who are wealthier. Although the risk of intrapartum stillbirth was lower in women who lived close to a high-quality facility compared with those living further away, it was not lower among wealthier women compared with women who were poorer (when adjusting for distance).

Women with more education had lower maternal mortality and fewer stillbirths (though not lower early neonatal mortality) than those with less education, suggesting that better-quality obstetric care is available in the area for this subgroup. We measured capability to provide good-quality care at the level of the facility. However, even facilities with good capability do not necessarily provide good care to all individuals. That educated women with better health knowledge were able to negotiate better care or were treated better because they could more easily relate to the providers is conceivable. The differing effects for education and wealth also highlight that these two aspects of socioeconomic status should be considered separately in analyses, and further work could seek to unravel the reasons why they have different effects.

Although we do not want to overinterpret the trend of higher maternal mortality among women who are wealthier and who lived closer to a facility of any level, nor the interactions by time period, it is conceivable that providers also undertake harmful practices,^47^ do harm by doing “too much, too soon”,^48^ or that unhygienic facility conditions foster the spread of infections.^49^, ^50^ After free health insurance was introduced for pregnant women in Ghana in 2008, facility birth surged.^33^ In situations of overcrowding and stagnating resources, as occurred then, quality of care, which was low overall in the study area,^40^ is likely to have been compromised. Facility birth might convey both benefits and harms, with the net effect on mortality depending on quality of care and on the case mix of women and babies who would benefit from skilled birth attendance, versus those who would not. Tew^14^ previously showed the fallacy of the statement “if it is accepted that confinement in hospital is safer for certain types of patients, where the risks are high, it must also be safer for cases where the risks are less”. The interplay of beneficial and harmful factors could explain the partially protective, partially detrimental effects of facility birth we found in our study area. Such an interpretation could also explain the heterogeneous findings seen in the literature in terms of the effect of facility birth on mortality.

Several rigorous large-scale studies did not find the expected harmful effect of longer distance to care on maternal or neonatal mortality, with effect estimates close to the null value or even showing protective effects, while observing a sharp decline in facility birth with increasing distance in the same populations.^22^, ^27^, ^29^ Hounton and co-workers^22^ speculate that this “may be due to the relatively poor capacity of health centres and district hospitals to deal with complications”. In several settings, mortality increased with distance from the closest hospital, but not with distance from the closest health centre,^26^, ^51^, ^52^ “consistent with evidence that these PHCs [primary health centres] are not well equipped to deal with complications”.^52^ In Malawi, where 92% of births were in facilities, neonatal mortality was found to be lower among babies born in a higher-quality facility than those born in a lower-quality facility, using differential distance between the closest facility and a high-quality facility as an instrumental variable.^53^

Studies on the effects of user-fee removals consistently find strong increases in facility birth, but few find significant reductions in mortality.^54^, ^55^ An evaluation of the Janani Suraksha Yojana (JSY) conditional cash-transfer programme in 284 districts in India found no association between district-level facility birth and maternal mortality in an adjusted model (with a trend in the wrong direction; ie, maternal mortality was higher in districts with higher proportions of facility birth). Randive and co-workers^20^ conclude that the “high institutional births that JSY has achieved are of themselves inadequate to reduce MMR [maternal mortality ratio]” and that “other factors including improved quality of care at institutions are required for intended effect”. While one study claimed an effect of JSY on neonatal mortality,^56^ supported by a replication study,^57^ another evaluation found the evidence insufficient and explained the absence of a mortality effect with the inability of lower-level facilities to manage life-threatening complications.^58^

A pooled DHS analysis of individual-level facility birth and early neonatal mortality found no overall association, and also found no association for birth in a hospital (OR 0·99, 95% CI 0·92–1·08), but a significantly increased mortality for birth in a health centre (OR 1·10, 1·06–1·14) in stratified analyses.^17^ These results are confounded by adverse selection, as is our analysis of individual-level facility birth (appendix, p 6). Nevertheless, that health centres attract more high-risk cases than hospitals seems unlikely, so this pattern cannot be explained by adverse selection alone and is consistent with deficient quality of care in health centres compared with hospitals.

Our study benefited from a large sample size and from a rigorous prospective pregnancy and mortality surveillance system in the context of two trials^35^, ^36^ with data both on maternal and perinatal mortality, including details on stillbirth timing. In addition, we collected data on several dimensions of quality of care through a health facility census, and we could study quality of care indirectly by using a policy change during the study period that led to overcrowding of facilities. These features make ours the most comprehensive dataset on the topic to date, enabling us to look more specifically at which type and quality of care saves lives at birth, and whose lives exactly are saved.

We explored a large range of alternative explanations for the absence of increase in mortality with distance to care despite the steep decline in facility birth with longer distance. Under-ascertainment of deaths among pregnant women in remote areas is the foremost concern. Furthermore, it is possible that some pregnancies were missed entirely, and more so in distant locations, and that mortality was higher in missed pregnancies. During the trials, pregnancies were recorded through monthly surveillance visits and mortality was followed up for all pregnancies, making under-reporting of deaths unlikely. Sensitivity analyses showed that results were not changed by excluding pregnancies with suboptimal surveillance quality. By contrast to cross-sectional surveys, such as the DHS, which collect data after birth, we can thus be confident that our results are not explained by selective under-reporting of deaths or misclassification of stillbirths and early neonatal deaths. Another potential explanation could be that women with high-risk pregnancies move closer to a health facility shortly before giving birth. To compute distances, we used women's regular place of residence as recorded during surveillance, not their immediate location before giving birth, so temporary movement cannot have affected our results.

Ours is an observational study, and although we adjusted for a wide range of potential confounders, and this adjustment made little difference to the findings, it is possible that unmeasured confounders influenced our results. However, only negative confounders could explain the absence of an effect on mortality (ie, factors that put those women at higher mortality risk who live closer to facilities or in clusters with higher levels of facility birth). Omission of positive confounders, such as antenatal-care attendance, by contrast, would overestimate the effects. A small number of potential negative confounders come to mind, mainly obesity and breastfeeding practices. Breastfeeding practices were, however, better among women living close to a facility than among those living further away. We did not measure body-mass index (BMI), but we adjusted for wealth quintile, which should capture obesity to some degree (BMI increases with wealth among women in Ghana).^59^ That our findings are explained by uncontrolled confounding is therefore unlikely.

A particular strength of this study is that we collected facility data on quality of care at birth in several dimensions, including more than 50 facility characteristics and a 20-point vignette assessing clinical competence, making our study the most comprehensive and rigorous quality assessment to date in such a large-scale setting.^40^ These data were collected in 2010 after the end of the data collection for the two trials.^35^, ^36^ This requires a strong assumption that relative quality of care remained constant during the entire observation period. Furthermore, some facilities may have opened or closed over time. Any misclassification of distance and quality of care will have biased the estimates towards the null value, and more so during the earlier two policy periods. Despite this potential bias, we observed very strong associations of distance with delivery in a facility and by caesarean section.

Our quality classification is based on theoretical capability to do certain functions. Few facilities reported that they were ready to perform CEmOC or EmNC functions and even fewer are likely to apply these to all women in a timely and appropriate manner.^40^ Although we could not measure quality as provided to individuals, we used clinical vignettes to assess provider competence in specific situations. The strong association of several quality measures with intrapartum stillbirth suggests that these measures captured quality of care at least to some degree. Nevertheless, our measures of quality have shortcomings and this might explain why access to higher-quality facilities was not associated with lower maternal and early neonatal mortality. However, wealth also did not show an association with any of the mortality outcomes, although women who were wealthier were more likely to deliver their babies in CEmOC facilities than women who were not. This result suggests that our null findings are not just due to limitations in our quality measure, but rather that even the wealthiest women in the best facilities did not receive care of sufficient quality to save lives.

Given the large sample size of this study, insufficient power was only a potential issue for maternal mortality. Considering any childbirth care (not specifying quality), maternal mortality actually decreased with increasing distance from care. Similarly, the point estimate for the association of maternal mortality and wealth was higher than 1, and point estimates for the associations between maternal mortality and distance to high-quality care were close to the null value. Insufficient power is thus an unlikely explanation for the absence of expected findings.

In terms of effect size, when comparing women who lived more than 20 km from a CEmOC facility to those who lived within 1 km, we observed a 50% absolute increase in the proportion of births in a CEmOC facility (from 10% to 60%) and a 27% relative reduction in intrapartum stillbirth risk (from 14·2 per 1000 deliveries to 10·4 per 1000 deliveries). Assuming equal risk distribution, we can calculate that an increase from 0% to 100% CEmOC facility birth would translate into a 54% decrease in intrapartum stillbirth. This reduction is smaller than the 75% reduction in intrapartum stillbirth for CEmOCs in the Lives Saved Tool, based on Yakoob's Delphi process.^8^, ^9^

In conclusion, we provided evidence that facility birth alone, in a setting with low facility capability^40^ and provider skill,^41^ does not confer any survival benefit for women or babies. Encouraging women to deliver in facilities that are unable to safely manage routine deliveries and complications might actually cause harm and be unethical.^12^, ^60^ The Ghanaian policy shift that increased facility birth without increasing resources did not confer benefit, and might have led to harm. Facility birth should only be recommended in facilities capable of providing emergency obstetric and newborn care and safe-guarding uncomplicated births.^12^ The focus needs to shift from increasing coverage with facility birth or skilled birth attendants, a “unidimensional and limited metric”,^12^ towards the complex challenge of strengthening health systems, training more health professionals, and improving quality of care at birth, and developing appropriate metrics to measure progress along this path.
