# Supplementary material for: Does facility birth reduce maternal and perinatal mortality in Brong Ahafo, Ghana? A secondary analysis using data on 119 244 pregnancies from two cluster-randomised controlled trials
Source: Lancet Glob Health. 2019 Jul 11;7(8):e1074–87. doi: 10.1016/S2214-109X(19)30165-2 (PMC6639244; doi:10.1016/S2214-109X(19)30165-2)
Supplement: Supplementary appendix [file mmc1.pdf]

THE LANCET
Global Health

Supplementary appendix

This appendix formed part of the original submission and has been peer reviewed. We post it as supplied by the authors.

Supplement to: Gabrysch S, Nesbitt RC, Schoeps A, et al. Does facility birth reduce maternal and perinatal mortality in Brong Ahafo, Ghana? A secondary analysis using data on 119 244 pregnancies from two cluster-randomised controlled trials. *Lancet Glob Health* 2019; **7:** e1074–87.

Supplementary appendix

Contents

1. Supplementary figures

Figure A1: Flowchart

Figure A2: Shape of distance relationships

Figure A3: Outcomes by birth year

2. Supplementary tables

Table A1: Adjusted associations of individual-level facility birth with seven mortality outcomes

Table A2: Crude associations of cluster-level facility birth, wealth, education and distances with nine health service use and mortality outcomes

Table A3: Adjusted associations of cluster-level facility birth, wealth, education and distances with nine health service use and mortality outcomes (underlying Figures 2 and 3)

Table A4: Interaction of time period with cluster-level facility birth, wealth, education and distances in their effect on four mortality outcomes (underlying Figure 4)

3. Sensitivity analyses

- Crude analysis in restricted sample (Table A5)
- Road distance and travel time (Table A6)
- Restricted sample to women with good pregnancy surveillance (Table A7)
- Three-level random effects model (Table A8)

1. Supplementary Figures

Figure A1: Flowchart of total numbers of pregnancies, deliveries and deaths in adjusted and unadjusted analyses

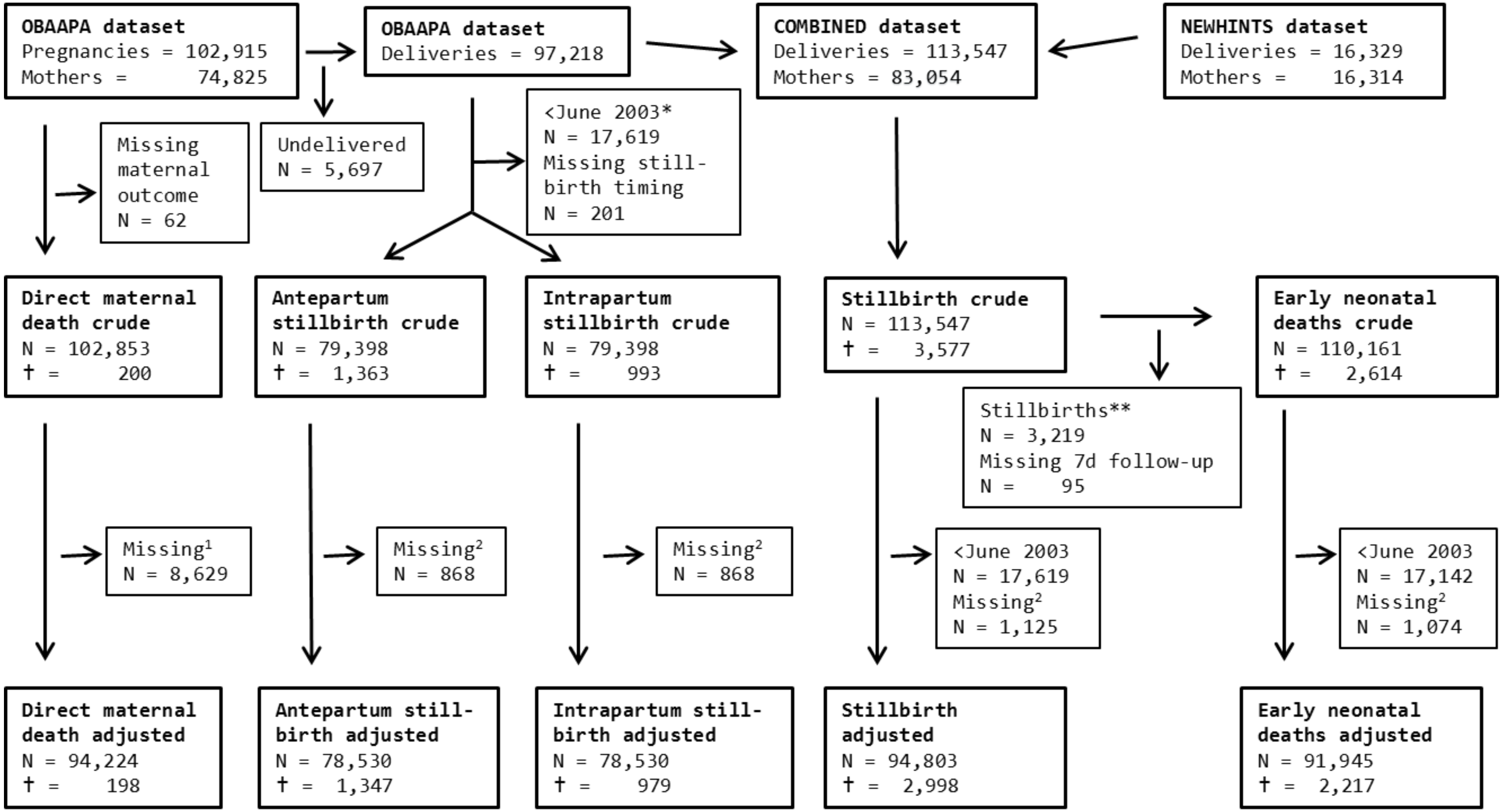

[1] Missings in religion, parity, ethnicity
[2] Missings in religion, parity, ethnicity, household wealth, education, multiple birth, occupation
* Timing of stillbirth was only collected after June 2003.
** Stillbirths minus those 286 multiple deliveries with a stillbirth where a twin/triplet baby was live born

Figure A2: Modeled effects of distance to quality care on service use and mortality

Estimated associations of distance to the closest facility providing Comprehensive Emergency Obstetric Care (CEmOC) with facility birth and Caesarean section (Panel A) and mortality outcomes (Panel B), based on the adjusted model with log-transformed distance.

Panel A:

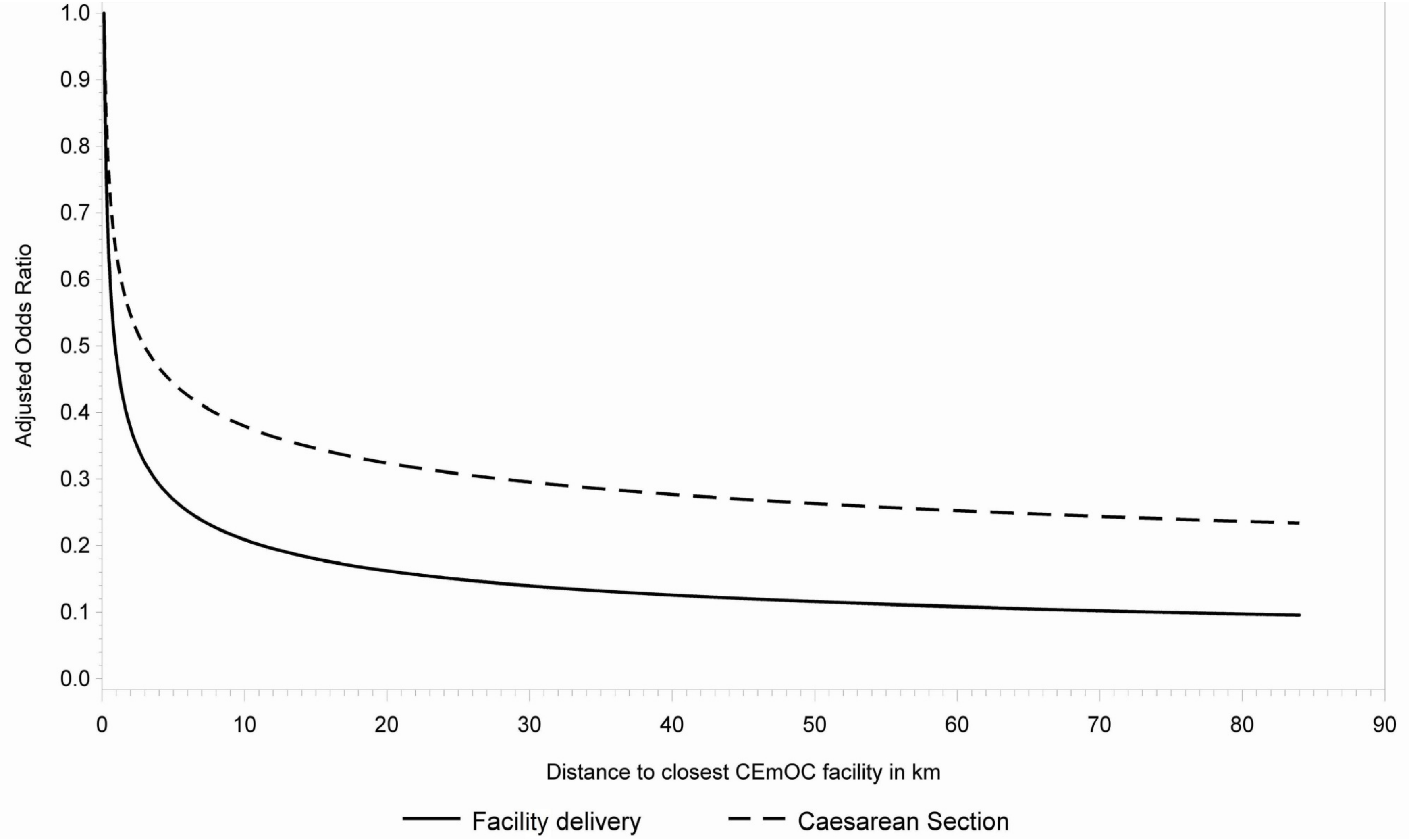

Panel B:

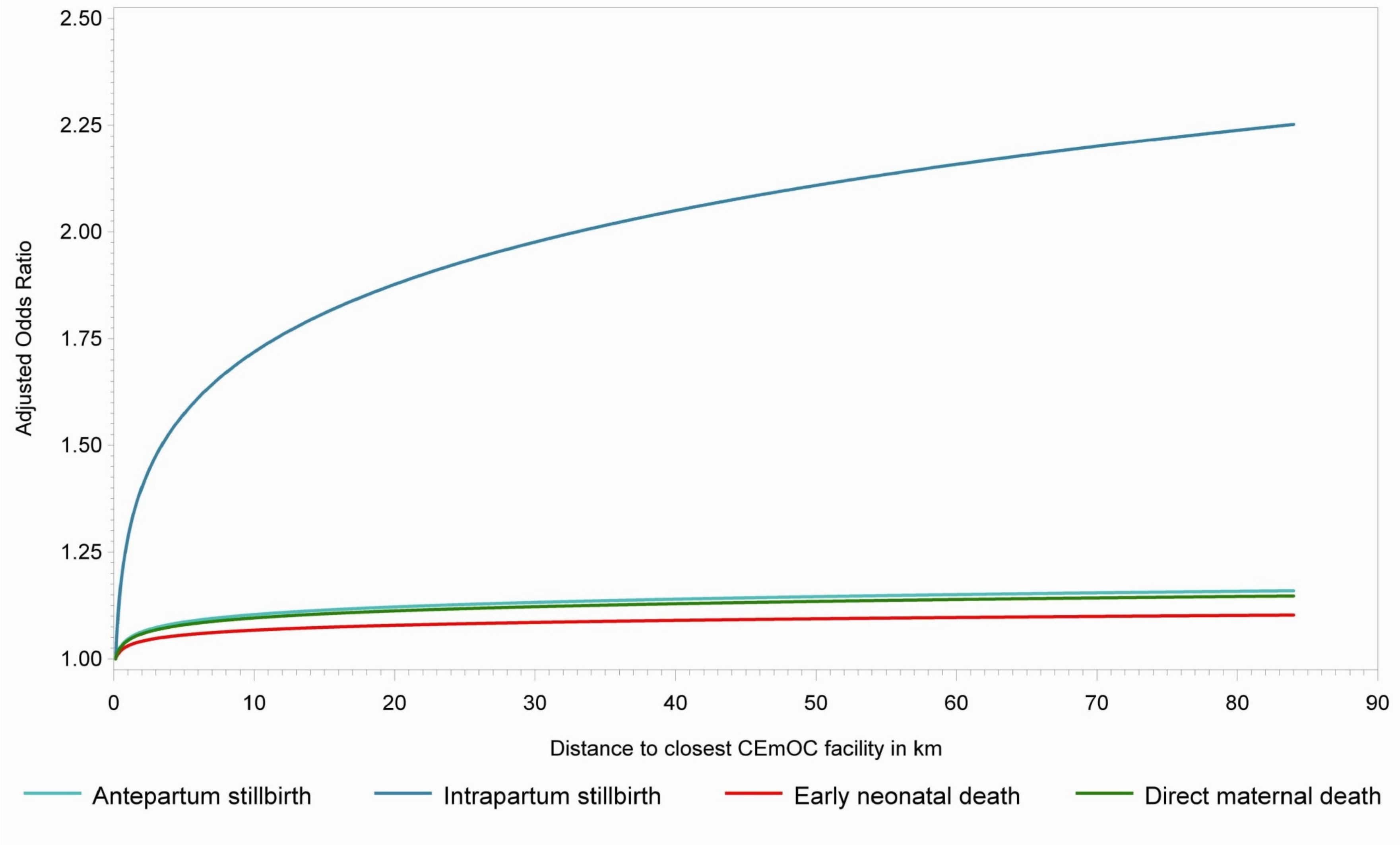

Adjusted Odds Ratio
2.50
2.25
2.00
1.75
1.50
1.25
1.00
0
10
20
30
40
50
60
70
80
90
Distance to closest CEmOC facility in km
Antepartum stillbirth
Intrapartum stillbirth
Early neonatal death
Direct maternal death

Figure A3: Health service use and mortalities by birth year

Facility birth and Caesarean section (right axis), and mortalities (left axis) are shown by year of birth. The grey shaded bars signify the distribution of deliveries over the years.

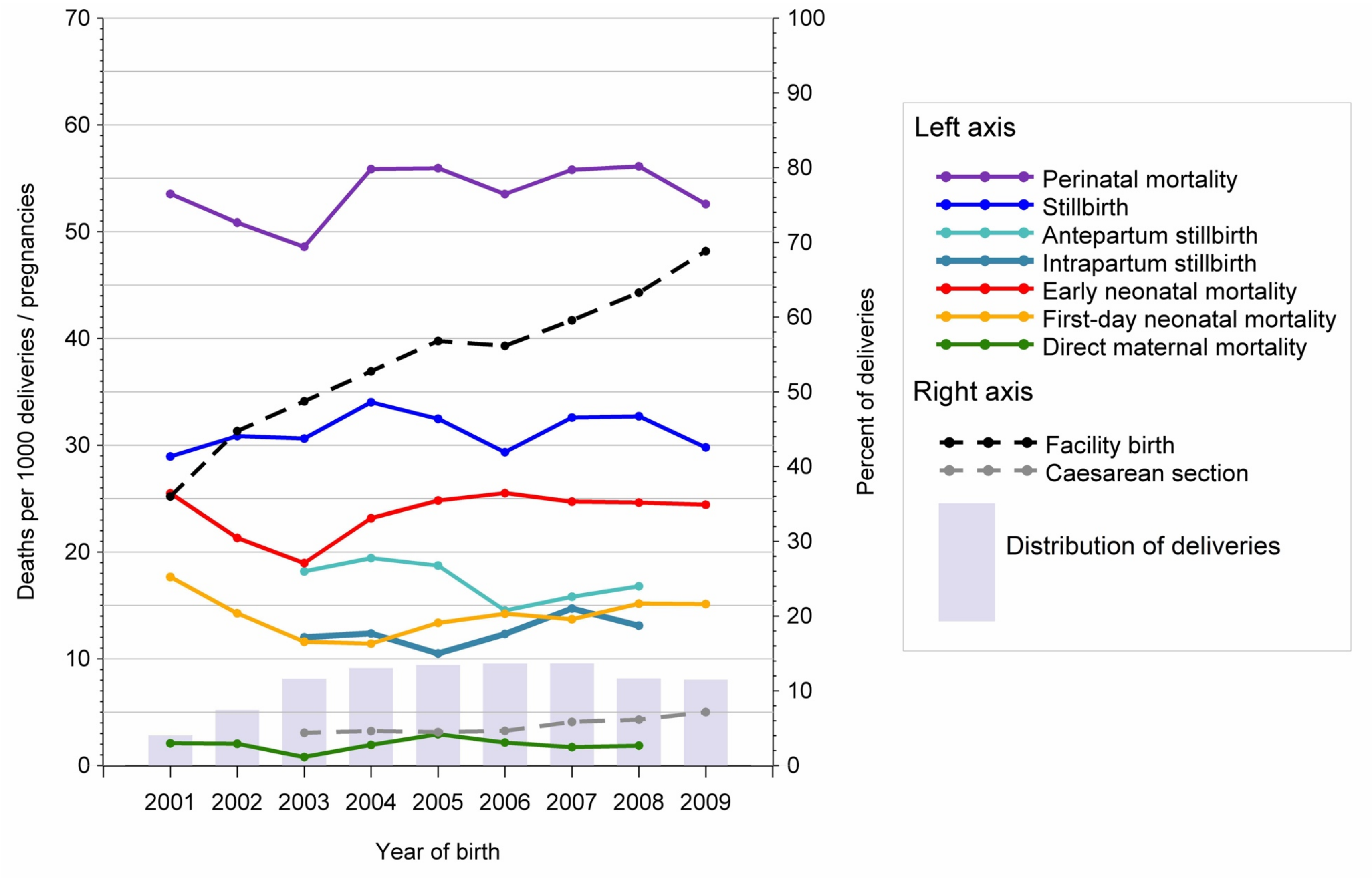

2. Supplementary Tables

Table A1: Adjusted associations of individual-level facility birth with seven mortality outcomes

Each line of results comes from a separate regression model of facility birth on an outcome, adjusting for confounders as specified in the methods section, using surveillance data from 2003 (for maternal mortality from 2000) to 2009 (for maternal mortality, antepartum and intrapartum stillbirth to 2008).

Exposure	Outcome	Odds Ratio	95% Confidence Interval		p-value	N
Facility delivery (of index birth)	Direct maternal mortality	1.36	0.81	2.28	0.243	89,365
	Perinatal mortality	1.67	1.56	1.80	<0.001	94,589
	Early neonatal mortality	1.43	1.29	1.58	<0.001	91,860
	First-day neonatal mortality	1.44	1.26	1.66	<0.001	91,860
	Stillbirth	1.81	1.65	1.98	<0.001	94,684
	Antepartum stillbirth	1.89	1.64	2.16	<0.001	78,424
	Intrapartum stillbirth	1.68	1.44	1.95	<0.001	78,424

Table A2: Crude associations of cluster-level facility birth, wealth, education and distances with nine health service use and mortality outcomes

Each line of results comes from a separate regression model of an exposure on an outcome, using surveillance data from 2000 to 2009 (for antepartum and intrapartum stillbirth from 2003-2008, for maternal mortality from 2000-2008).

Exposure	Outcome	Odds Ratio	95% Confidence Interval		p-value	N
Cluster-level facility birth (per 20% increase)	Facility birth	2.36	2.30	2.41	<0.001	98,084
	Caesarean section	1.39	1.34	1.45	<0.001	93,610
	Direct maternal mortality	1.13	1.02	1.26	0.025	86,607
	Perinatal mortality	1.00	0.98	1.02	0.976	98,141
	Early neonatal mortality	1.02	0.99	1.05	0.204	95,262
	First-day neonatal mortality	1.04	1.00	1.08	0.042	95,262
	Stillbirth	0.98	0.96	1.01	0.241	98,236
	Antepartum stillbirth	0.99	0.95	1.04	0.688	77,786
	Intrapartum stillbirth	0.96	0.91	1.01	0.079	77,786
Household wealth (per quintile)	Facility birth	1.75	1.72	1.77	<0.001	105,026
	Caesarean section	1.36	1.33	1.40	<0.001	94,719
	Direct maternal mortality	1.18	1.04	1.34	0.013	93,620
	Perinatal mortality	1.02	1.00	1.04	0.080	105,197
	Early neonatal mortality	1.03	1.00	1.07	0.035	102,146
	First-day neonatal mortality	1.05	1.01	1.09	0.019	102,146
	Stillbirth	1.01	0.98	1.03	0.659	105,292
	Antepartum stillbirth	1.04	0.99	1.08	0.097	78,702
	Intrapartum stillbirth	0.95	0.90	1.00	0.034	78,702
Mother's education (per level)	Facility birth	1.66	1.63	1.69	<0.001	105,423
	Caesarean section	1.39	1.35	1.44	<0.001	95,028
	Direct maternal mortality	0.88	0.76	1.02	0.093	94,059
	Perinatal mortality	1.02	0.99	1.05	0.132	105,638

	Early neonatal mortality	1.06	1.02	1.11	0.003	102,563
	First-day neonatal mortality	1.11	1.05	1.17	<0.001	102,563
	Stillbirth	0.99	0.95	1.02	0.437	105,733
	Antepartum stillbirth	1.01	0.95	1.06	0.833	78,869
	Intrapartum stillbirth	0.93	0.87	1.00	0.045	78,869
Distance (logarithmic) to closest facility offering						
Any childbirth care	Facility birth	0.54	0.53	0.56	<0.001	113,125
	Caesarean section	0.80	0.77	0.84	<0.001	95,397
	Direct maternal mortality	0.87	0.78	0.96	0.007	102,853
	Perinatal mortality	0.98	0.96	1.01	0.150	113,452
	Early neonatal mortality	0.97	0.94	1.00	0.076	110,161
	First-day neonatal mortality	0.95	0.91	0.99	0.012	110,161
	Stillbirth	0.99	0.97	1.02	0.652	113,547
	Antepartum stillbirth	0.99	0.94	1.04	0.611	79,398
	Intrapartum stillbirth	1.05	0.99	1.11	0.080	79,398
Comprehensive Emergency Obstetric Care	Facility birth	0.55	0.53	0.57	<0.001	113,125
	Caesarean section	0.68	0.66	0.71	<0.001	95,397
	Direct maternal mortality	0.95	0.87	1.04	0.286	102,853
	Perinatal mortality	1.01	0.99	1.03	0.386	113,452
	Early neonatal mortality	0.99	0.95	1.02	0.363	110,161
	First-day neonatal mortality	0.98	0.94	1.01	0.228	110,161
	Stillbirth	1.03	1.00	1.06	0.064	113,547
	Antepartum stillbirth	0.98	0.93	1.03	0.330	79,398
	Intrapartum stillbirth	1.13	1.07	1.20	<0.001	79,398
Emergency Newborn Care	Facility birth	0.66	0.63	0.68	<0.001	113,125
	Caesarean section	0.73	0.70	0.76	<0.001	95,397

	Direct maternal mortality	0.94	0.85	1.04	0.241	102,853
	Perinatal mortality	1.02	0.99	1.04	0.183	113,452
	Early neonatal mortality	0.99	0.96	1.03	0.626	110,161
	First-day neonatal mortality	1.00	0.96	1.04	0.944	110,161
	Stillbirth	1.04	1.01	1.07	0.014	113,547
	Antepartum stillbirth	1.01	0.96	1.06	0.807	79,398
	Intrapartum stillbirth	1.12	1.06	1.18	<0.001	79,398
High-quality routine care	Facility birth	0.56	0.54	0.58	<0.001	113,125
	Caesarean section	0.71	0.68	0.74	<0.001	95,397
	Direct maternal mortality	0.92	0.84	1.02	0.108	102,853
	Perinatal mortality	1.00	0.98	1.02	0.958	113,452
	Early neonatal mortality	0.97	0.94	1.00	0.061	110,161
	First-day neonatal mortality	0.96	0.92	1.00	0.027	110,161
	Stillbirth	1.02	0.99	1.05	0.218	113,547
	Antepartum stillbirth	0.98	0.93	1.03	0.403	79,398
	Intrapartum stillbirth	1.09	1.03	1.15	0.003	79,398
Vignette score >12	Facility birth	0.61	0.59	0.63	<0.001	113,125
	Caesarean section	0.71	0.68	0.74	<0.001	95,397
	Direct maternal mortality	0.96	0.87	1.05	0.356	102,853
	Perinatal mortality	1.01	0.98	1.03	0.568	113,452
	Early neonatal mortality	0.98	0.95	1.01	0.107	110,161
	First-day neonatal mortality	0.97	0.93	1.00	0.062	110,161
	Stillbirth	1.03	1.00	1.06	0.039	113,547
	Antepartum stillbirth	0.97	0.92	1.02	0.205	79,398
	Intrapartum stillbirth	1.13	1.07	1.19	<0.001	79,398

Table A3: Adjusted associations of cluster-level facility birth, wealth, education and distances with nine health service use and mortality outcomes (underlying Figures 2 and 3)

Each line of results comes from a separate regression model of an exposure on an outcome, adjusting for confounders as specified in the methods section, using surveillance data from 2003 (for maternal mortality from 2000) to 2009 (for maternal mortality, antepartum and intrapartum stillbirth to 2008).

Exposure	Outcome	Odds Ratio	95% Confidence Interval		p-value	N
Cluster-level facility birth (per 20% increase)	Facility birth	1.82	1.77	1.87	<0.001	93,141
	Caesarean section	1.16	1.12	1.21	<0.001	92,730
	Direct maternal mortality	1.10	0.94	1.28	0.235	94,224
	Perinatal mortality	0.98	0.95	1.01	0.120	93,164
	Early neonatal mortality	0.99	0.95	1.03	0.716	90,434
	First-day neonatal mortality	1.00	0.95	1.05	0.986	90,434
	Stillbirth	0.97	0.94	1.00	0.075	93,259
	Antepartum stillbirth	0.96	0.91	1.01	0.127	76,992
	Intrapartum stillbirth	0.96	0.90	1.02	0.176	76,992
Household wealth (per quintile)	Facility birth	1.45	1.42	1.47	<0.001	94,684
	Caesarean section	1.13	1.09	1.16	<0.001	94,183
	Direct maternal mortality	1.16	0.97	1.38	0.098	94,224
	Perinatal mortality	1.01	0.99	1.04	0.349	94,708
	Early neonatal mortality	1.00	0.96	1.05	0.837	91,945
	First-day neonatal mortality	1.00	0.95	1.06	0.962	91,945
	Stillbirth	1.02	0.98	1.06	0.358	94,803
	Antepartum stillbirth	1.02	0.97	1.08	0.462	78,530
	Intrapartum stillbirth	1.00	0.94	1.07	0.973	78,530
Mother's education (per level)	Facility birth	1.24	1.21	1.27	<0.001	94,684
	Caesarean section	1.13	1.09	1.18	<0.001	94,183
	Direct maternal mortality	0.69	0.56	0.85	<0.001	94,224

	Perinatal mortality	0.96	0.93	1.00	0.039	94,708
	Early neonatal mortality	0.99	0.93	1.04	0.652	91,945
	First-day neonatal mortality	1.07	0.99	1.15	0.078	91,945
	Stillbirth	0.94	0.89	0.98	0.010	94,803
	Antepartum stillbirth	0.94	0.87	1.01	0.078	78,530
	Intrapartum stillbirth	0.94	0.87	1.03	0.190	78,530
Distance (logarithmic) to closest facility offering						
Any childbirth care	Facility birth	0.62	0.60	0.64	<0.001	94,684
	Caesarean section	0.90	0.87	0.94	<0.001	94,183
	Direct maternal mortality	0.88	0.78	1.00	0.042	94,224
	Perinatal mortality	1.00	0.97	1.03	0.992	94,708
	Early neonatal mortality	0.99	0.95	1.03	0.611	91,945
	First-day neonatal mortality	0.97	0.93	1.02	0.273	91,945
	Stillbirth	1.01	0.97	1.04	0.716	94,803
	Antepartum stillbirth	1.01	0.96	1.06	0.666	78,530
	Intrapartum stillbirth	1.03	0.98	1.09	0.251	78,530
Comprehensive Emergency Obstetric Care	Facility birth	0.69	0.67	0.72	<0.001	94,684
	Caesarean section	0.80	0.77	0.83	<0.001	94,183
	Direct maternal mortality	1.01	0.89	1.15	0.871	94,224
	Perinatal mortality	1.04	1.01	1.07	0.006	94,708
	Early neonatal mortality	1.02	0.98	1.06	0.389	91,945
	First-day neonatal mortality	1.01	0.96	1.06	0.717	91,945
	Stillbirth	1.06	1.02	1.09	0.003	94,803
	Antepartum stillbirth	1.02	0.96	1.08	0.481	78,530
	Intrapartum stillbirth	1.13	1.06	1.21	<0.001	78,530
Emergency Newborn Care	Facility birth	0.79	0.76	0.82	<0.001	94,684

	Caesarean section	0.85	0.81	0.88	<0.001	94,183
	Direct maternal mortality	0.99	0.88	1.12	0.926	94,224
	Perinatal mortality	1.05	1.03	1.08	<0.001	94,708
	Early neonatal mortality	1.03	0.99	1.06	0.152	91,945
	First-day neonatal mortality	1.03	0.99	1.08	0.169	91,945
	Stillbirth	1.07	1.04	1.10	<0.001	94,803
	Antepartum stillbirth	1.06	1.01	1.12	0.024	78,530
	Intrapartum stillbirth	1.10	1.03	1.17	0.002	78,530
High-quality routine care	Facility birth	0.69	0.66	0.71	<0.001	94,684
	Caesarean section	0.83	0.79	0.86	<0.001	94,183
	Direct maternal mortality	0.99	0.87	1.12	0.814	94,224
	Perinatal mortality	1.02	1.00	1.05	0.105	94,708
	Early neonatal mortality	1.00	0.96	1.03	0.804	91,945
	First-day neonatal mortality	0.98	0.94	1.03	0.433	91,945
	Stillbirth	1.04	1.00	1.07	0.040	94,803
	Antepartum stillbirth	1.02	0.97	1.08	0.370	78,530
	Intrapartum stillbirth	1.07	1.01	1.14	0.027	78,530
Vignette score >12	Facility birth	0.74	0.71	0.76	<0.001	94,684
	Caesarean section	0.80	0.77	0.84	<0.001	94,183
	Direct maternal mortality	1.02	0.90	1.15	0.771	94,224
	Perinatal mortality	1.03	1.00	1.06	0.027	94,708
	Early neonatal mortality	1.01	0.97	1.05	0.724	91,945
	First-day neonatal mortality	1.00	0.95	1.05	0.916	91,945
	Stillbirth	1.05	1.01	1.08	0.008	94,803
	Antepartum stillbirth	1.01	0.95	1.06	0.811	78,530
	Intrapartum stillbirth	1.12	1.05	1.19	<0.001	78,530

Table A4: Interaction of time period with cluster-level facility birth, wealth, education and distances in their effect on four mortality outcomes (underlying Figure 4)

Each Odds Ratio (OR) and 95% Confidence Interval (CI) come from a separate regression model of an exposure on an outcome, with an interaction term for time period. The main effects are shown stratified by time period (calculated using the interaction terms) as well as the interaction p-values. Models are adjusted for confounders as specified in the methods section and based on surveillance data from 2003 to 2009.

				Time period 6/2003 - 3/2005				Time period 4/2005 - 06/2008				interaction	Time period 07/2008 - 12/2009				interaction
	Exposure	Outcome	N	OR	95% CI		p-value	OR	95% CI		p-value	p-value	OR	95% CI		p-value	p-value
Cluster-level facility birth(per 20% increase)		Perinatal mortality	93164	0.96	0.92	1.00	0.051	0.97	0.93	1.00	0.034	0.819	1.05	1.00	1.11	0.045	0.003
		Early neonatal mortality	90434	0.99	0.93	1.05	0.701	0.97	0.93	1.02	0.287	0.698	1.06	0.99	1.15	0.112	0.117
		First-day neonatal mortality	90434	0.94	0.86	1.02	0.130	0.99	0.93	1.05	0.773	0.245	1.12	1.02	1.24	0.019	0.003
		Stillbirth	93259	0.95	0.90	1.00	0.038	0.96	0.92	1.00	0.058	0.642	1.04	0.97	1.11	0.264	0.020
Household wealth (per quintile)		Perinatal mortality	94708	0.99	0.94	1.03	0.571	1.00	0.97	1.04	0.891	0.534	1.09	1.03	1.14	0.001	0.002
		Early neonatal mortality	91945	0.99	0.92	1.06	0.686	0.98	0.93	1.03	0.492	0.920	1.10	1.02	1.19	0.013	0.020
		First-day neonatal mortality	91945	0.92	0.84	1.02	0.099	1.00	0.93	1.07	0.929	0.139	1.10	1.00	1.21	0.058	0.006
		Stillbirth	94803	0.99	0.93	1.05	0.668	1.01	0.97	1.06	0.520	0.392	1.07	1.00	1.14	0.048	0.050
Mother's education (per level)		Perinatal mortality	94708	0.93	0.88	0.99	0.027	0.97	0.93	1.02	0.224	0.253	0.97	0.91	1.04	0.403	0.374
		Early neonatal mortality	91945	0.98	0.89	1.08	0.688	1.00	0.93	1.07	0.961	0.744	0.97	0.87	1.07	0.525	0.826
		First-day neonatal mortality	91945	1.08	0.95	1.23	0.223	1.09	0.99	1.19	0.075	0.963	1.02	0.89	1.16	0.798	0.476
		Stillbirth	94803	0.90	0.83	0.97	0.008	0.95	0.89	1.01	0.073	0.270	0.97	0.88	1.06	0.468	0.208
Distance (logarithmic) to closest facility offering																	
	Any childbirth care	Perinatal mortality	94708	1.01	0.97	1.05	0.655	1.02	0.99	1.06	0.172	0.599	0.93	0.89	0.98	0.007	0.014
		Early neonatal mortality	91945	0.99	0.93	1.06	0.800	1.01	0.97	1.06	0.612	0.590	0.94	0.87	1.01	0.078	0.226
		First-day neonatal mortality	91945	1.02	0.94	1.12	0.587	0.99	0.93	1.05	0.628	0.447	0.89	0.81	0.98	0.016	0.028
		Stillbirth	94803	1.02	0.96	1.07	0.547	1.03	0.99	1.07	0.205	0.739	0.94	0.88	1.00	0.051	0.049
	Comprehensive Emergency Obstetric Care	Perinatal mortality	94708	1.07	1.02	1.11	0.004	1.05	1.02	1.09	0.004	0.541	0.98	0.94	1.03	0.502	0.007
		Early neonatal mortality	91945	1.04	0.97	1.10	0.282	1.03	0.98	1.08	0.224	0.895	0.97	0.90	1.04	0.346	0.125
		First-day neonatal mortality	91945	1.05	0.97	1.15	0.235	1.01	0.95	1.07	0.701	0.410	0.96	0.88	1.04	0.304	0.094
		Stillbirth	94803	1.08	1.02	1.14	0.006	1.07	1.02	1.11	0.005	0.635	1.00	0.94	1.06	0.975	0.043
	Emergency Newborn Care	Perinatal mortality	94708	1.09	1.05	1.14	<0.001	1.07	1.03	1.10	<0.001	0.389	0.96	0.92	1.01	0.106	<0.001
		Early neonatal mortality	91945	1.05	0.99	1.12	0.131	1.07	1.02	1.12	0.005	0.655	0.90	0.84	0.96	0.003	<0.001
		First-day neonatal mortality	91945	1.08	0.99	1.19	0.086	1.07	1.01	1.14	0.023	0.885	0.90	0.82	0.98	0.018	0.003
		Stillbirth	94803	1.11	1.05	1.17	<0.001	1.07	1.02	1.11	0.002	0.215	1.02	0.95	1.08	0.643	0.030
	High-quality routine care	Perinatal mortality	94708	1.06	1.02	1.11	0.008	1.02	0.99	1.06	0.169	0.154	0.97	0.93	1.02	0.287	0.006
		Early neonatal mortality	91945	1.00	0.94	1.06	0.998	1.01	0.96	1.06	0.787	0.860	0.96	0.90	1.03	0.293	0.408
		First-day neonatal mortality	91945	1.01	0.93	1.10	0.832	0.98	0.93	1.04	0.599	0.614	0.94	0.86	1.03	0.197	0.261
		Stillbirth	94803	1.09	1.03	1.15	0.002	1.03	0.99	1.07	0.187	0.067	0.98	0.92	1.05	0.586	0.009
	Vignette score >12	Perinatal mortality	94708	1.04	1.00	1.09	0.055	1.04	1.01	1.08	0.013	0.965	0.99	0.94	1.04	0.663	0.086
		Early neonatal mortality	91945	1.01	0.95	1.07	0.798	1.02	0.98	1.07	0.335	0.669	0.97	0.90	1.04	0.334	0.343
		First-day neonatal mortality	91945	1.03	0.95	1.12	0.429	1.00	0.94	1.06	0.983	0.477	0.95	0.87	1.04	0.263	0.153
		Stillbirth	94803	1.06	1.00	1.11	0.039	1.06	1.01	1.10	0.010	1.000	1.01	0.95	1.07	0.753	0.235

3. Sensitivity analyses

1) Crude analysis in restricted sample

Multivariable analyses were performed in a restricted sample, including only observations from 2003 (for all outcomes except maternal mortality) and observations without missing values in the adjustment variables (99% after 2003). To rule out bias by complete case analysis and by restricting to observations after 2003, additional crude analyses were performed in the restricted sample used for adjusted analyses, and results were compared with results of the crude analysis in the complete sample.
Results from crude analyses in the restricted sample used for adjusted analyses (Table A5) were similar to those in the full sample (compare Table A2).

Table A5: Crude associations of cluster-level facility birth, wealth, education and distances with nine health service use and mortality outcomes (in the subset of observations used for adjusted analyses in Table A3)

Each line of results comes from a separate regression model of an exposure on an outcome, using surveillance data from 2000 to 2009 (for antepartum and intrapartum stillbirth from 2003-2008, for maternal mortality from 2000-2008)

Exposure	Outcome	Odds Ratio	95% Confidence Interval		p-value	N
Cluster-level facility birth (per 20% increase)	Facility birth	2.34	2.29	2.40	<0.001	93,141
	Caesarean section	1.40	1.34	1.45	<0.001	92,730
	Direct maternal mortality	1.13	0.97	1.32	0.107	80,945
	Perinatal mortality	1.00	0.97	1.02	0.706	93,164
	Early neonatal mortality	1.02	0.98	1.05	0.331	90,434
	First-day neonatal mortality	1.04	1.00	1.08	0.069	90,434
	Stillbirth	0.98	0.95	1.01	0.134	93,259
	Antepartum stillbirth	0.99	0.95	1.03	0.595	76,992
	Intrapartum stillbirth	0.95	0.90	1.00	0.046	76,992
Household wealth (per quintile)	Facility birth	1.77	1.74	1.80	<0.001	94,684
	Caesarean section	1.36	1.33	1.40	<0.001	94,183
	Direct maternal mortality	1.12	0.98	1.29	0.105	82,546
	Perinatal mortality	1.02	0.99	1.04	0.175	94,708
	Early neonatal mortality	1.03	1.00	1.06	0.088	91,945
	First-day neonatal mortality	1.05	1.01	1.09	0.021	91,945
	Stillbirth	1.00	0.97	1.03	0.923	94,803

	Antepartum stillbirth	1.04	0.99	1.08	0.097	78,530
	Intrapartum stillbirth	0.95	0.90	0.99	0.029	78,530
Mother's education (per level)	Facility birth	1.67	1.64	1.70	<0.001	94,684
	Caesarean section	1.39	1.35	1.44	<0.001	94,183
	Direct maternal mortality	0.95	0.79	1.16	0.641	82,546
	Perinatal mortality	1.01	0.98	1.04	0.436	94,708
	Early neonatal mortality	1.05	1.01	1.10	0.022	91,945
	First-day neonatal mortality	1.11	1.05	1.17	<0.001	91,945
	Stillbirth	0.98	0.94	1.01	0.235	94,803
	Antepartum stillbirth	1.01	0.95	1.07	0.788	78,530
	Intrapartum stillbirth	0.93	0.87	1.00	0.037	78,530
Distance (logarithmic) to closest facility offering						
Any childbirth care	Facility birth	0.54	0.52	0.55	<0.001	94,684
	Caesarean section	0.80	0.76	0.83	<0.001	94,183
	Direct maternal mortality	0.83	0.71	0.97	0.020	82,546
	Perinatal mortality	0.99	0.97	1.01	0.462	94,708
	Early neonatal mortality	0.98	0.95	1.01	0.176	91,945
	First-day neonatal mortality	0.96	0.92	1.00	0.038	91,945
	Stillbirth	1.00	0.97	1.03	0.844	94,803
	Antepartum stillbirth	0.99	0.94	1.03	0.599	78,530
	Intrapartum stillbirth	1.05	1.00	1.11	0.060	78,530
Comprehensive Emergency Obstetric Care	Facility birth	0.56	0.54	0.58	<0.001	94,684
	Caesarean section	0.68	0.65	0.71	<0.001	94,183
	Direct maternal mortality	0.91	0.78	1.06	0.216	82,546
	Perinatal mortality	1.02	0.99	1.04	0.157	94,708
	Early neonatal mortality	1.00	0.97	1.03	0.862	91,945

	First-day neonatal mortality	0.98	0.94	1.02	0.327	91,945
	Stillbirth	1.03	1.00	1.06	0.033	94,803
	Antepartum stillbirth	0.98	0.94	1.03	0.512	78,530
	Intrapartum stillbirth	1.13	1.07	1.20	<0.001	78,530
					<0.001	
Emergency Newborn Care	Facility birth	0.65	0.63	0.68	<0.001	94,684
	Caesarean section	0.73	0.70	0.76	<0.001	94,183
	Direct maternal mortality	0.94	0.81	1.09	0.438	82,546
	Perinatal mortality	1.03	1.00	1.05	0.028	94,708
	Early neonatal mortality	1.00	0.97	1.04	0.803	91,945
	First-day neonatal mortality	1.01	0.97	1.05	0.699	91,945
	Stillbirth	1.05	1.02	1.08	<0.001	94,803
	Antepartum stillbirth	1.01	0.97	1.07	0.580	78,530
	Intrapartum stillbirth	1.12	1.06	1.18	<0.001	78,530
High-quality routine care	Facility birth	0.56	0.54	0.58	<0.001	94,684
	Caesarean section	0.71	0.68	0.74	<0.001	94,183
	Direct maternal mortality	0.87	0.75	1.01	0.061	82,546
	Perinatal mortality	1.01	0.98	1.03	0.666	94,708
	Early neonatal mortality	0.98	0.95	1.01	0.263	91,945
	First-day neonatal mortality	0.96	0.93	1.00	0.072	91,945
	Stillbirth	1.02	0.99	1.05	0.183	94,803
	Antepartum stillbirth	0.99	0.94	1.03	0.555	78,530
	Intrapartum stillbirth	1.09	1.03	1.15	0.002	78,530
Vignette score >12	Facility birth	0.61	0.59	0.63	<0.001	94,684
	Caesarean section	0.71	0.68	0.74	<0.001	94,183
	Direct maternal mortality	0.93	0.80	1.08	0.337	82,546
	Perinatal mortality	1.01	0.99	1.04	0.267	94,708

Early neonatal mortality	0.99	0.96	1.02	0.599	91,945
First-day neonatal mortality	0.98	0.94	1.01	0.199	91,945
Stillbirth	1.03	1.00	1.06	0.047	94,803
Antepartum stillbirth	0.98	0.93	1.03	0.346	78,530
Intrapartum stillbirth	1.13	1.07	1.19	<0.001	78,530

2) Road distance and travel time

We performed analyses using road distance and walking time in hours, calculated assuming a travel speed of 4 km per hour for travel along the road and a speed of 2 km per hour across land where no road was available (Nesbitt et al. 2014, ref. 39). For comparability with straight-line distance, both measures were also log-transformed.
Results when using road distance or walking time (Table A6) were similar to those using straight-line distance (compare Table A3).

Table A6: Adjusted associations of two road distances and two walking-time measures with nine health service use and mortality outcomes

Each line of results comes from a separate regression model of an exposure on an outcome, adjusting for confounders as specified in the methods section, using surveillance data from 2003 (for maternal mortality from 2000) to 2009 (for maternal mortality, antepartum and intrapartum stillbirth to 2008).

Exposure	Outcome	Odds Ratio	95% Confidence Interval		p-value	N
Road distance (logarithmic) to closest facility offering						
Any childbirth care	Facility birth	0.62	0.60	0.64	<0.001	94,684
	Caesarean section	0.92	0.88	0.96	<0.001	94,183
	Direct maternal mortality	0.90	0.79	1.02	0.088	94,224
	Perinatal mortality	0.99	0.97	1.02	0.660	94,708
	Early neonatal mortality	0.99	0.95	1.03	0.581	91,945
	First-day neonatal mortality	0.97	0.93	1.02	0.283	91,945
	Stillbirth	1.00	0.96	1.03	0.847	94,803
	Antepartum stillbirth	1.01	0.96	1.06	0.730	78,530
	Intrapartum stillbirth	1.02	0.96	1.08	0.585	78,530
Comprehensive Emergency Obstetric Care	Facility birth	0.69	0.67	0.73	<0.001	94,684
	Caesarean section	0.78	0.74	0.82	<0.001	94,183
	Direct maternal mortality	1.03	0.89	1.19	0.697	94,224
	Perinatal mortality	1.04	1.01	1.07	0.022	94,708
	Early neonatal mortality	1.02	0.97	1.06	0.457	91,945
	First-day neonatal mortality	1.01	0.95	1.06	0.837	91,945
	Stillbirth	1.05	1.01	1.09	0.013	94,803
	Antepartum stillbirth	1.02	0.96	1.08	0.614	78,530
	Intrapartum stillbirth	1.13	1.05	1.21	<0.001	78,530

Walking time (per hour) to closest facility offering							
	Any childbirth care	Facility birth	0.654	0.634	0.675	<0.001	94,684
		Caesarean section	0.882	0.844	0.922	<0.001	94,183
		Direct maternal mortality	0.926	0.810	1.058	0.258	94,224
		Perinatal mortality	0.994	0.970	1.020	0.659	94,708
		Early neonatal mortality	0.994	0.959	1.031	0.763	91,945
		First-day neonatal mortality	0.998	0.952	1.046	0.928	91,945
		Stillbirth	0.995	0.965	1.027	0.770	94,803
		Antepartum stillbirth	1.006	0.958	1.057	0.806	78,530
		Intrapartum stillbirth	1.017	0.965	1.072	0.533	78,530
	Comprehensive Emergency Obstetric Care	Facility birth	0.958	0.947	0.968	<0.001	94,684
		Caesarean section	0.929	0.915	0.944	<0.001	94,183
		Direct maternal mortality	1.032	0.993	1.073	0.110	94,224
		Perinatal mortality	1.010	1.002	1.018	0.020	94,708
		Early neonatal mortality	1.007	0.995	1.019	0.267	91,945
		First-day neonatal mortality	1.001	0.985	1.016	0.950	91,945
		Stillbirth	1.012	1.002	1.023	0.024	94,803
		Antepartum stillbirth	1.000	0.984	1.018	0.958	78,530
		Intrapartum stillbirth	1.022	1.005	1.040	0.012	78,530

3) Restricted sample to women with good pregnancy surveillance

To assess possible (differential) under-reporting of deaths, we performed an analysis only including pregnancies that were recorded before delivery (100% in the *Newhints* trial) or with at least one surveillance visit during the last 3 months of pregnancy (97% in the *ObaapaVita* trial).
Results when excluding deliveries with suboptimal surveillance (Table A7) were similar to those in the full sample (compare Table A3).

Table A7: Adjusted associations of cluster-level facility birth, wealth, education and distances with 8 health service use and mortality outcomes in the subset with good surveillance (pregnancy visit in 90 days before birth)

Each line of results comes from a separate regression model of an exposure on an outcome, adjusting for confounders as specified in the methods section, using surveillance data from 2003 to 2009 (for antepartum and intrapartum stillbirth to 2008).

Exposure	Outcome	Odds Ratio	95% Confidence Interval		p-value	N
Cluster-level facility birth (per 20% increase)	Facility birth	1.85	1.80	1.90	<0.001	90,388
	Caesarean section	1.16	1.12	1.21	<0.001	89,988
	Perinatal mortality	0.98	0.95	1.00	0.091	90,407
	Early neonatal mortality	0.99	0.95	1.03	0.717	87,731
	First-day neonatal mortality	1.01	0.96	1.06	0.779	87,731
	Stillbirth	0.97	0.93	1.00	0.053	90,502
	Antepartum stillbirth	0.96	0.91	1.01	0.127	74,240
	Intrapartum stillbirth	0.95	0.90	1.01	0.100	74,240
Household wealth (per quintile)	Facility birth	1.44	1.42	1.47	<0.001	91,905
	Caesarean section	1.13	1.09	1.16	<0.001	91,415
	Perinatal mortality	1.01	0.98	1.04	0.355	91,925
	Early neonatal mortality	1.00	0.96	1.05	0.954	89,216
	First-day neonatal mortality	1.00	0.95	1.06	0.992	89,216
	Stillbirth	1.02	0.98	1.06	0.305	92,020
	Antepartum stillbirth	1.02	0.96	1.08	0.536	75,752
	Intrapartum stillbirth	1.01	0.95	1.08	0.777	75,752
Mother's education (per level)	Facility birth	1.24	1.21	1.27	<0.001	91,905

		Caesarean section	1.12	1.08	1.17	<0.001	91,415
		Perinatal mortality	0.96	0.92	1.00	0.043	91,925
		Early neonatal mortality	0.98	0.93	1.04	0.600	89,216
		First-day neonatal mortality	1.06	0.98	1.14	0.132	89,216
		Stillbirth	0.94	0.89	0.99	0.014	92,020
		Antepartum stillbirth	0.94	0.87	1.01	0.082	75,752
		Intrapartum stillbirth	0.95	0.87	1.04	0.246	75,752
Distance (logarithmic) to closest facility offering							
	Any childbirth care	Facility birth	0.62	0.60	0.64	<0.001	91,905
		Caesarean section	0.90	0.87	0.94	<0.001	91,415
		Perinatal mortality	1.00	0.98	1.03	0.819	91,925
		Early neonatal mortality	0.99	0.96	1.03	0.757	89,216
		First-day neonatal mortality	0.97	0.93	1.02	0.231	89,216
		Stillbirth	1.01	0.98	1.04	0.624	92,020
		Antepartum stillbirth	1.02	0.96	1.07	0.560	75,752
		Intrapartum stillbirth	1.04	0.98	1.10	0.222	75,752
	Comprehensive Emergency Obstetric Care	Facility birth	0.69	0.66	0.72	<0.001	91,905
		Caesarean section	0.80	0.76	0.83	<0.001	91,415
		Perinatal mortality	1.04	1.01	1.07	0.006	91,925
		Early neonatal mortality	1.02	0.97	1.06	0.465	89,216
		First-day neonatal mortality	1.00	0.96	1.06	0.865	89,216
		Stillbirth	1.06	1.02	1.10	0.002	92,020
		Antepartum stillbirth	1.02	0.97	1.08	0.449	75,752
		Intrapartum stillbirth	1.14	1.07	1.21	<0.001	75,752
	Emergency Newborn Care	Facility birth	0.78	0.75	0.81	<0.001	91,905
		Caesarean section	0.84	0.81	0.88	<0.001	91,415

	Perinatal mortality	1.06	1.03	1.08	<0.001	91,925
	Early neonatal mortality	1.03	0.99	1.07	0.118	89,216
	First-day neonatal mortality	1.03	0.98	1.08	0.201	89,216
	Stillbirth	1.07	1.04	1.11	<0.001	92,020
	Antepartum stillbirth	1.07	1.01	1.12	0.015	75,752
	Intrapartum stillbirth	1.10	1.04	1.17	0.002	75,752
High-quality routine care	Facility birth	0.69	0.66	0.71	<0.001	91,905
	Caesarean section	0.82	0.79	0.86	<0.001	91,415
	Perinatal mortality	1.02	0.99	1.05	0.120	91,925
	Early neonatal mortality	0.99	0.95	1.03	0.632	89,216
	First-day neonatal mortality	0.98	0.93	1.02	0.302	89,216
	Stillbirth	1.04	1.00	1.08	0.033	92,020
	Antepartum stillbirth	1.02	0.97	1.08	0.405	75,752
	Intrapartum stillbirth	1.08	1.01	1.15	0.018	75,752
Vignette score >12	Facility birth	0.73	0.71	0.76	<0.001	91,905
	Caesarean section	0.80	0.77	0.83	<0.001	91,415
	Perinatal mortality	1.03	1.00	1.06	0.033	91,925
	Early neonatal mortality	1.00	0.97	1.04	0.889	89,216
	First-day neonatal mortality	0.99	0.95	1.04	0.758	89,216
	Stillbirth	1.05	1.01	1.09	0.006	92,020
	Antepartum stillbirth	1.01	0.95	1.06	0.823	75,752
	Intrapartum stillbirth	1.12	1.06	1.19	<0.001	75,752

4) Three-level random effects model

To account for clustering of outcomes due to multiple deliveries of the same mother, we used three-level regression models with mother at level 2 and village/suburb at level 3. Results when using three-level regression models (Table A8) were similar to those using two-level models (compare Table A2).
We used two-level models as our main analysis because the running time for three-level models was extremely long.

Table A8: Crude associations of cluster-level facility birth, wealth and two distances with three mortality outcomes using a three-level random effects model

Each line of results comes from a separate regression model of an exposure on an outcome, using surveillance data from 2000 to 2009 (for antepartum and intrapartum stillbirth from 2003-2008, for maternal mortality from 2000-2008).

Exposure	Outcome	Odds Ratio	95% Confidence Interval		p-value	N
Cluster-level facility birth (per 20% increase)	Early neonatal mortality	1.02	0.99	1.06	0.209	95,262
	Antepartum stillbirth	0.99	0.95	1.04	0.700	77,786
	Intrapartum stillbirth	0.95	0.91	1.00	0.074	77,786
Household wealth (per quintile)	Early neonatal mortality	1.03	1.00	1.07	0.037	102,146
	Antepartum stillbirth	1.04	0.99	1.08	0.099	78,702
	Intrapartum stillbirth	0.94	0.90	1.00	0.032	78,702
Distance (logarithmic) to closest facility offering						
Any childbirth care	Early neonatal mortality	0.97	0.94	1.00	0.075	110,161
	Antepartum stillbirth	0.99	0.94	1.04	0.603	79,398
	Intrapartum stillbirth	1.05	0.99	1.11	0.079	79,398
Comprehensive Emergency Obstetric Care	Early neonatal mortality	0.99	0.95	1.02	0.357	110,161
	Antepartum stillbirth	0.98	0.93	1.03	0.324	79,398
	Intrapartum stillbirth	1.14	1.07	1.20	<0.001	79,398
